# Global Spectrum of Copy Number Variations Reveals Genome Organizational Plasticity and Proposes New Migration Routes

**DOI:** 10.1371/journal.pone.0121846

**Published:** 2015-04-24

**Authors:** Avinash M. Veerappa, Sangeetha Vishweswaraiah, Kusuma Lingaiah, Megha Murthy, Raviraj V. Suresh, Dinesh S. Manjegowda, Nallur B. Ramachandra

**Affiliations:** 1 Genetics and Genomics Lab, Department of Studies in Zoology, University of Mysore, Manasagangotri, Mysore-06, Karnataka, India; 2 NUCSER, KS Hegde Medical Academy, Nitte University, Mangalore-18, Karnataka, India; Harvard Medical School, UNITED STATES

## Abstract

Global spectrum of CNVs is required to catalog variations to provide a high-resolution on the dynamics of genome-organization and human migration. In this study, we performed genome-wide genotyping using high-resolution arrays and identified 44,109 CNVs from 1,715 genomes across 12 populations. The study unraveled the force of independent evolutionary dynamics on genome-organizational plasticity across populations. We demonstrated the use of CNV tool to study human migration and identified a second major settlement establishing new migration routes in addition to existing ones.

## Introduction

Human genome is complex in its organization with defined structural entities, which include heterochromatin, euchromatin, centromeres and telomeres, which are conserved in most eukaryotic species [1]. Cataloguing the nature and pattern of variations in these structures are fundamental in understanding human genetic diversity. Human genome shows diverse array of variations ranging from single base to several Mbs. They are single nucleotide polymorphisms (SNPs), insertions-Deletions (inDels), segmental duplications (SDs) and copy number variations (CNVs), present at different levels and frequencies [2]. SNPs for long were thought to be the predominant form of genomic variation accounting for much normal phenotypic variation [3]. However, widespread presence of CNVs in normal individuals seems to be a significant contributor to the phenotypic variation [4–9] and affects more nucleotides per genome than SNPs. CNVs are a form of structural variation in the genome ranging from 1kb to several Mbs which perturbs the normal biological balance of dualistic allele segmental state which include duplications and deletions found in all humans.

Numerous CNVs are being identified with various genome analysis platforms, including array comparative genomic hybridization (aCGH), SNP genotyping, and next-generation sequencing [10–11]. Presence of varying frequencies of CNVs across and within continents [12–13] indicates the diversity in CNV distribution among different populations. This genetic diversity in humans affects both disease and normal phenotypic variation. Presence of CNVs alters the transcriptional and translational levels of overlapping or nearby genes by disrupting the coding structure or by altering gene dosage thereby conferring differential susceptibility to complex diseases [14–18].

Both low and high resolution CNV studies have been performed across control population cohorts since 2003, majorly covering Africa, America, Europe, China, Tibet, Taiwan, India, Germany and Finland [12, 19–26]. High-resolution CNV studies will also provide an insight on the history of human migration, expansion, and adaptation. Therefore, in the current study, we report the global spectrum of CNV distribution and genome plasticity, and also propose new migration routes.

## Materials and Methods

For this study, a total of 1767 individuals (1715 after quality check) from 12 different populations including 43 normal members from India (randomly selected 12 families residing in Karnataka), 270 HapMap samples covering CEU (CEPH collection), CHB (Han Chinese in Beijing, China), JPT (Japanese in Tokyo, Japan) and YRI (Yoruba in Ibadan, Nigeria) populations, 31 Tibetan samples, 155 Chinese samples, 471 of Ashkenazi Jews replicate 1, 482 of Ashkenazi Jews replicate 2, 204 individuals from Taiwan, 64 from Australia and 47 from New World population (Totonacs and Bolivians), were selected. Five milliliter of EDTA blood was collected from each member of the Indian study group and genomic DNA was extracted using Promega Wizard Genomic DNA purification kit (Promega Corporation, Madison, WI, United States of America). The isolated DNA was quantified by Bio-photometer and gel electrophoresis. Written informed consent was obtained from all sample donors and this research was approved by the University of Mysore Institutional Human Ethics review committee (IHEC).

The 270 individuals sample data from the four populations was obtained from the International HapMap Consortium [19]. The raw, unprocessed data from Affymetrix Genome Wide SNP 6.0 array (Affymetrix, Inc. Santa Clara, CA, United States of America) for the 31 individuals from Tibet population and the remaining 6 populations were obtained from the ArrayExpress Archive of the European Bioinformatics Institute. The CNV data from these populations had not been analyzed by the original authors and had remained largely unexplored as these were the control subsets of the experiment. The global share of CNVs was obtained by the total number of CNVs of that population over the total number of CNVs of all populations. The CNVs identified in this study are highly consistent, because of the higher stringency adopted in both the selection and validation of CNVs using multiple algorithms. Since the 1kb to 100kb CNVs showed maximum signal to noise ratio, we restricted our investigations to a CNV size limit of only >100kb. The comparative analysis of the same ethnic group (Ashkenazi Jews I and II) as a negative control, show very similar data points with negligible deviations hence validating our data. The following datasets were obtained from the ArrayExpress archive with the accession numbers E-GEOD-21661, E-GEOD-29851, E-GEOD-30481, E-GEOD-15826, E-GEOD-23636, E-GEOD-23201, E-GEOD-33355 and E-GEOD-33356. Data has been made publicly accessible through the FigShare public repository. The dataset can be accessed at http://dx.doi.org/10.6084/m9.figshare.1320382

### Genotyping

The human genome build hg18 was used as a reference genome. Genome-wide genotyping was performed using an Affymetrix Genome-wide Human SNP Array 6.0 chip and Affymetrix CytoScan High-Density (HD) Array having 1.8 million and 2.6 million combined SNP and CNV markers with the median inter- marker distance of 500–600 bases (Affymetrix, Inc. Santa Clara, CA, USA). These chips provide maximum panel power and the highest physical coverage of the genome [47–50]. Genotyping quality was assessed using Affymetrix Genotyping Console Software (Affymetrix, Inc. Santa Clara, CA, USA). Copy Number Analysis Method offers two types of segmenting methods, univariate and multivariate. These methods are based on the same algorithm, but use different criteria for determining cut-points denoting CNV boundaries. CNVs >100kb were only included for the analysis ruling out the 1kb-99kb range CNVs, as we believe including these will only create more background noise with just too many false-positives and eliminating those CNVs with false-positives can be misleading.

### Algorithms for Copy Number state calling

#### BirdSuite (v2)

BirdSuite [51] is a suite originally developed to detect known common Copy Number Polymorphisms (CNPs) based on prior knowledge, as well as to discover rare CNVs, from Affymetrix SNP 6.0 array data. To do this, it incorporates two main methods; the “Birdsuite” algorithms and the “Canary” [52]. The Birdsuite algorithm uses a Hidden Markov model (HMM) approach to find regions of variable copy number in a sample. For the HMM, the hidden state is the true copy number of the individual’s genome and the observed states are the normalized intensity measurements of each array probe. CNV calls from the Canary and Birdsuite algorithms were collated for each sample, and kept as long as they met the following criteria: i) Birdsuite calls with a log_10_ of odds (LOD) score (Odds Ratio) ≥10 (corresponding to an approximate False Discovery Rate of ~5%), ii) Birdsuite calls with copy number states other than 2 were retained; iii) Canary CNP calls with CN states different from the population mode were retained.

#### Canary

CNP analysis was performed using the Canary algorithm. Canary was developed by the Broad Institute for making copy number state calls in genomic regions with CNPs. Canary algorithm computes a single intensity summary statistic using a subset of manually selected probes within the CNP region. The intensity summaries are compared in aggregate across all samples to intensity summaries previously observed in training data to assign a copy number state call.

#### CNVFinder

CNVFinder developed at the Welcome Trust Sanger Institute uses a dynamic, multiple-threshold based approach to allow robust classification of copy number changes in data of varying qualities. This algorithm makes two main assumptions i) that the majority of data points are normally distributed around a log_2_ ratio of zero, and ii) that data points falling outside of the centralized log_2_ ratio distribution are representative of a difference in copy number between test and reference genome.

### Genotyping Console

After processing CEL files and the Birdseed to call genotypes, we used the Genotyping Console (GTC v.3.0.2) to detect CNVs from the Affymetrix 6.0 array for samples that passed initial QCs. The default parameters of >1Kb size and >5 probes in this algorithm were used.

### Data Analysis

Genome-wide CNV study was carried out using SVS Golden Helix Ver. 7.2 [53] and Affymetrix Genotyping Console software as prescribed in their manuals [47–50] (Affymetrix, Inc. Santa Clara, CA, USA). Eigenstrat method was used to avoid possibility of spurious associations resulting from population stratification. Bonferroni correction was employed for multiple testing and the corrected data were then used for CNV testing. Bonferroni methods for population data genotyped on the Affymetrix 6.0 platform was α = 0.05 thresholds between 1 × 10^–7^ and 7 × 10^–8^. We adopted higher stringency screening methods for CNVs to overcome possible unequal sample size affects.

Analyzing the collated data from both BirdSuite and Canary algorithms increased the stringency on those meeting the CNP calls with a log_10_ of odds score greater than or equal to 10 corresponding to a False Discovery Rate of ~5%. All SNPs that were called using Birdseed v2algorithm had a Quality Control (QC) call rate of >97% across individuals. All the subjects and members with SNPs that passed SNP QC procedures were entered into the CNV analysis. Filters were set for ID call rates for the overall SNPs to identify IDs with poor quality DNA, if any. The CNV calls were generated using the Canary algorithm. In AGCS, contrast QC has to be >0.4 to be included in the CNV analyses. In this study, contrast QC observed was >2.5 across all samples showing a robust strength. To control for the possibility of spurious or artifact CNVs, we used the EIGENSTRAT approach of Price et al., [54]. This method derives the principal components of the correlations among gene variants and corrects for those correlations in the testing. We removed 52 individuals from the study group because they were extreme outliers on one or more significant EIGENSTRAT axes and further dropped 543 CNVs in the members selected for the study for not meeting the required QC measures. CNVs were considered validated when there was a reciprocal overlap of 50% or greater with the reference set. Though the Jaccard statistic is sensitive to the number of CNVs called by each algorithm (ideally each two algorithms would detect similar number of CNV calls), the relative values between the different comparisons of algorithms/platform/site are very informative. All the overlap analyses performed have handled losses and gains separately except when otherwise stated, and were conducted hierarchically. The calls from the algorithms that were called in both were not considered; instead, they were collated so that the relative values between the different comparisons of algorithms/platform/site are still very informative.

### Generation of CNV map

The shared map of 379 CNVs across all chromosomes (except 22^nd^ chromosome) was generated using the Circos software package [55].

### Calculating Genetic Distance using CNV distribution

Genetic similarities and differences were characterized using CNV genotypes between individuals of all the populations. Genetic distance between populations can be defined as the average of pairwise genotype differences between individuals from all the populations. Based on this, we constructed a phylogenetic tree by Neighbor-joining and UPGMA and the consensus phylogenetic tree was constructed using MEGA 5.1. MEGA was used to infer and draw phylogenetic trees, estimating divergence times, inferring ancestral relativity, and for testing evolutionary hypotheses. CNV sharing analysis was conducted by counting the number of CNV sharing regions among populations. The threshold value for the minimum number of populations a CNV is shared was set to 6 to increase the stringency and to determine relatively old and new CNVs. Phylogenetic trees were constructed using unweighted pair group method (UPGMA). UPGMA was used to construct phylogenetic trees using the rates of CNV similarities and differences. For this purpose the number of observed CNV breakpoints was compared across each populations. UPGMA employs a sequential clustering algorithm, in which local topological relationships are identified in order of similarity, and the phylogenetic tree is built in a stepwise manner.

### Breakpoint validations

In order to validate the presence of the CNVs, PCR amplification was performed on four recurring CNV breakpoints on 500 randomly chosen individuals from India. Chimeric primers flanking normal and deleted/duplicated sequence were designed to bind only to the CNV breakpoint regions. Samples that do not contain these specific CNVs, would fail to amplify. The primers used for amplifying the breakpoint regions are—AGGTCTGTTATGTGGCTGAGCCGCA on 3q29 for breakpoints 195276060bp- 195446910bp; ACTCTAGCCAACACATCCTCTGCGC on 15q14 for 34695310bp- 34857998bp; GAGTAAAGAAACAAAGGCCATCT on 21q11.2 for 14594223bp- 15101046bp; AGGGATCCACCCCCTGGCTGTGGA on 16p13.11 for 16377650bp— 16635603bp at annealing temperatures of 64°C, 73°C, 62°C, and 71°C respectively using DreamTaq polymerase in Kyratec PCR System (Kyratec, Australia). All PCR products were analyzed on 1% agarose gels and documented with a Vilber Lourmat Imaging system (Vilber Lourmat, France).

## Results

### CNVs across populations

**Fig 1:**
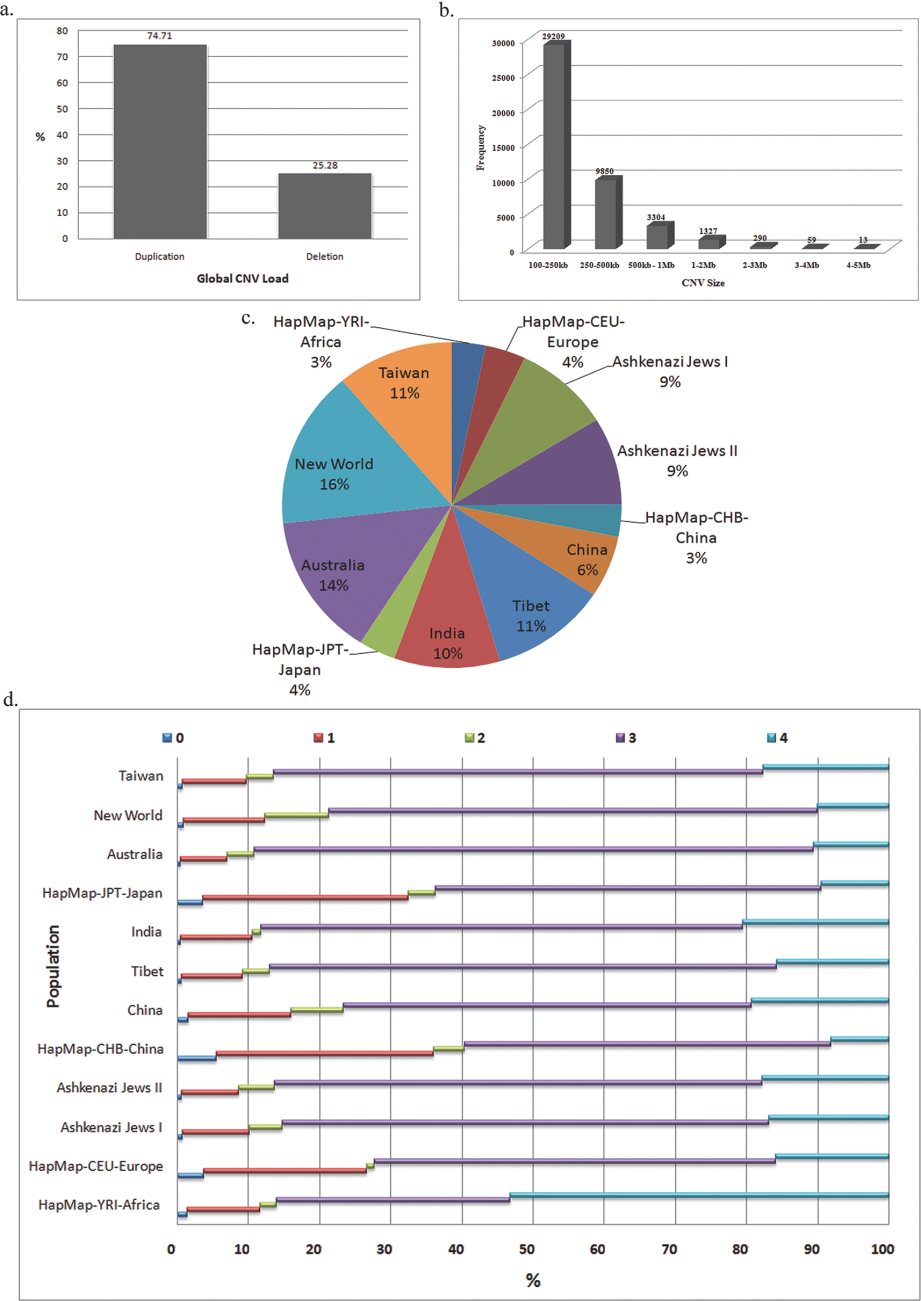
Distribution of CNV load and size across populations. a) Percent of Global CNV load across all populations. A total of 44109 CNVs across the genome showing the frequencies of gain polymorphisms (74.71%) and the loss polymorphisms (25.28%). b) Frequency of CNV size burden across populations. Bars indicate the number of CNVs for 7 different size ranges. 66.3%, 22.3%, 7.5%, 3%, 0.65% and 0.1% were found in the size range of 100-250kb, 250-500kb, 500kb-1Mb, 1-2Mb, 2-3Mb and 3-4Mb size respectively. c) Pie chart indicating percent of global share of CNVs identified in 12 populations. Each color represents an individual population and the percent of CNVs identified in that population. d) CN states of 0, 1, 2, 3 and 4 for all duplication and deletion CNVs across all populations where the elevated steps indicate the CN states in the increasing order. CHB, CEU, JPT, China and Africa showed a mean of 4.2% in the CN = 0 state, CHB, JPT, and CEU showed a mean of 27.4% while Africa seemed balancing in the CN = 1 allele state. CN = 2 state of sex chromosomes was found to be under heavy burden in New World, China and Ashkenazi Jews with a mean of 7.1% and least in CEU and India with a mean of 1.1%. CN = 3 state was observed high in all populations at 55–71% but was balanced in Africa (30%). CN = 4 state varied and Africa showed the highest concentration (53%), while Australia, New World, JPT and CHB showed the lowest dual duplication CN state (9%) compared to the remaining populations (~15–20%).

We identified a total of 44109 CNVs from 1715 genomes analyzed in 12 sample criteria understudy (Table 1). CNVs were observed across all populations, and no one individual was spared from the burden load of CNVs in the genome. A majority of CNVs were identified in the range of 5 to >50 CNVs for each individual and was found varying across population based on ethnicity. Duplicated CNVs were significantly higher than deletions (Fig 1A). Of the total CNVs analyzed, about 66.3%, 22.3% and 7.5% were found in the size range of 100-250kb, 250-500kb and 500kb-1Mb respectively indicating that a significant portion (88.6%) is within 500kb. The CNVs considerably declined as the size of the variation increased, with the least CNV count in the 4-5Mb range (Fig 1B). New World showed the highest CNV counts with higher duplications than deletions, while YRI and CHB showed the lowest CNV events, Fig 1C and Table 1. Individuals in the New World population (13.05 ± 6.5) showed the highest CNV size in the diploid genome, followed by Taiwan (10.24 ± 5.1) while CHB (3.02 ± 1.5) showed the lowest CNV size whereas the Asian and European populations did not show consistent pattern. Global CNV size estimates showed an individual carrying a variation of 7 ± 3Mb (Table 1).

**Table 1:** Genome-wide distribution of duplication and deletion CNVs across 12 populations.

Populations			Duplications (in mean)				Deletions (in mean)			CNV*			
										Count			Total
	IndividualsAssessed	Number	X^2^	P value	Size (Mb)	Number	X^2^	P value	Size (Mb)	%	X^2^	P value	Size (Mb)
**HapMap-YRI-Africa**	90	5.35	2.15	0.014	1.96	4.3	0.56	0.46	1.23	9.74	0.53	0.46	3.07±1.5
**HapMap-CEU-Europe**	90	5.77	1.81	0.178	1.76	5.9	1.456	0.227	1.73	11.76	0.086	0.76	5.95±3.0
**Ashkenazi Jews I**	464	21.11	2.94	0.086	6.10	6.72	2.023	0.154	1.72	27.8	5.174	0.0229	7.83±3.9
**Ashkenazi Jews II**	480	20.8	2.78	0.095	6.20	5.53	1.217	0.269	1.38	26.41	4.32	0.036	7.32±3.6
**HapMap-CHB-China**	44	3.91	3.62	0.05	1.12	5.79	1.383	0.239	1.37	9.70	0.543	0.461	3.02±1.5
**China**	155	12.75	0.083	0.773	4.67	5.89	1.449	0.228	1.32	18.64	0.918	0.61	6.19±3.09
**Tibet**	31	27.00	6.39	0.011	8.45	6.91	2.161	0.141	1.63	33.90	9.065	0.0026	5.5±2.7
**India**	38	23.18	4.06	0.043	7.10	7.52	2.617	0.105	1.87	30.70	6.947	0.008	8.9±2.2
**HapMap-JPT-Japan**	45	4.88	2.57	0.108	1.90	5.84	1.416	0.234	1.53	10.73	0.261	0.609	3.76±1.8
**Australia**	53	35.21	12.24	0.00046	8.60	7.83	2.855	0.091	2.02	43.03	15.78	0.00007	9.82±4.9
**New World**	41	33.61	11.03	0.0008	1.10	13.9	8.075	0.004	4.10	47.51	19.347	0.00001	13.05±6.5
**Taiwan**	184	26.16	5.85	0.015	7.95	8.22	3.162	0.075	2.31	34.39	9.402	0.0021	10.24±5.1

*CNV column includes both duplication and deletion events.

### Copy Number (CN) State

CN states (Fig 1D) for all duplication and deletion CNVs were assessed based on the 0, 1, 2, 3 and 4 states, where the numerical value represents its corresponding allele presence in the genome. HapMap and China showed increased loss of both allelic segments (CN = 0) in the genome. Further, CHB, JPT, and CEU showed a loss of one allele state in the other parts of the genome, however, Africa seemed moderate with the CN = 1 allele state. Nearly all populations (except Africa) showed a high number of CN = 3 autosomal duplication. The CN = 4 state was seen with a marked difference with Africa showing the highest concentration, while Australia, New World, JPT and CHB showed the lowest CN state. CN state in the sex chromosomes (CN = 2) was found to be under heavy burden in New World, China and Ashkenazi Jews, while CEU and India showed the least burden.

### Recurring and Rare CNVs

**Fig 2:**
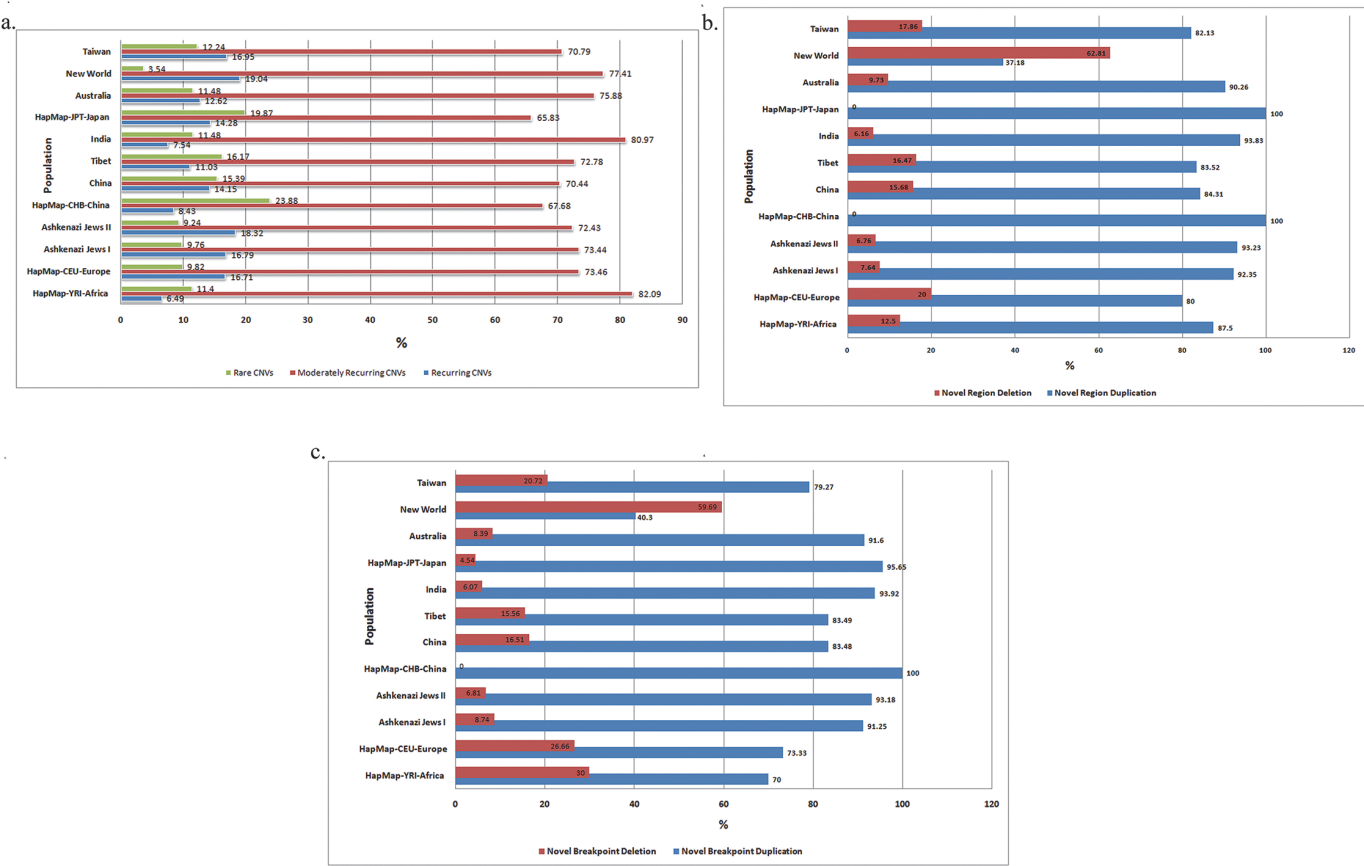
Nature of occurrence and location of CNVs. Each cluster represents an individual population. a) Frequencies of rare, moderately recurring and recurring CNVs are represented in the first second and third bars respectively. Recurring CNVs were found ranging from 6–18% in the entire study population and moderately recurring CNVs at 66–82%. Rare CNVs showed varied distribution across populations and were high in CHB, JPT, India, China, and Tibet, with a mean of 17% while New World showed the least (2%). b) Percent of novel regions found across populations are represented wherein red bars indicate deletions and blue bars indicate duplications. Duplication CNVs were higher with a mean of 85.3% than deletion CNVs (mean of 14.6%). c) Percent of novel breakpoints wherein red bars indicate deletions and blue bars indicate duplications. Novel breakpoint duplications (>70–100%) were greater than deletions (59.6%).

The CNVs identified from all the populations were grouped into classes of CNVs with frequency of >70% regarded as recurring CNVs, while, CNVs which were <5% in a population were regarded as rare CNVs and those CNVs which were neither belonging to these groups (5–70%), were considered as moderately recurring CNVs. The occurrences of moderately recurring CNVs were greater than the recurring CNVs. Most of the Asian countries showed slightly lesser number of recurring CNVs (~10%), but the remaining countries showed increased frequencies (>15%). China showed the highest frequency (22%) for rare CNVs, while New World showed the least (2%) across all populations (Fig 2A).

Duplication CNVs in novel regions were typically more than deletion CNVs (Fig 2B). India, Ashkenazi Jews and Australia showed substantial load of CNVs in novel regions, compared to New World which showed the least load of CNVs, while the remaining populations remained stable. Novel breakpoints were similarly found increased (>70–100%) in duplicated regions than deletion regions except for New World. Interestingly, CHB showed no novel breakpoint deletions, and nearly all the breakpoints identified were the duplication breakpoints (Fig 2C).

### Minor Allele Frequency of CNV Breakpoints

**Fig 3:**
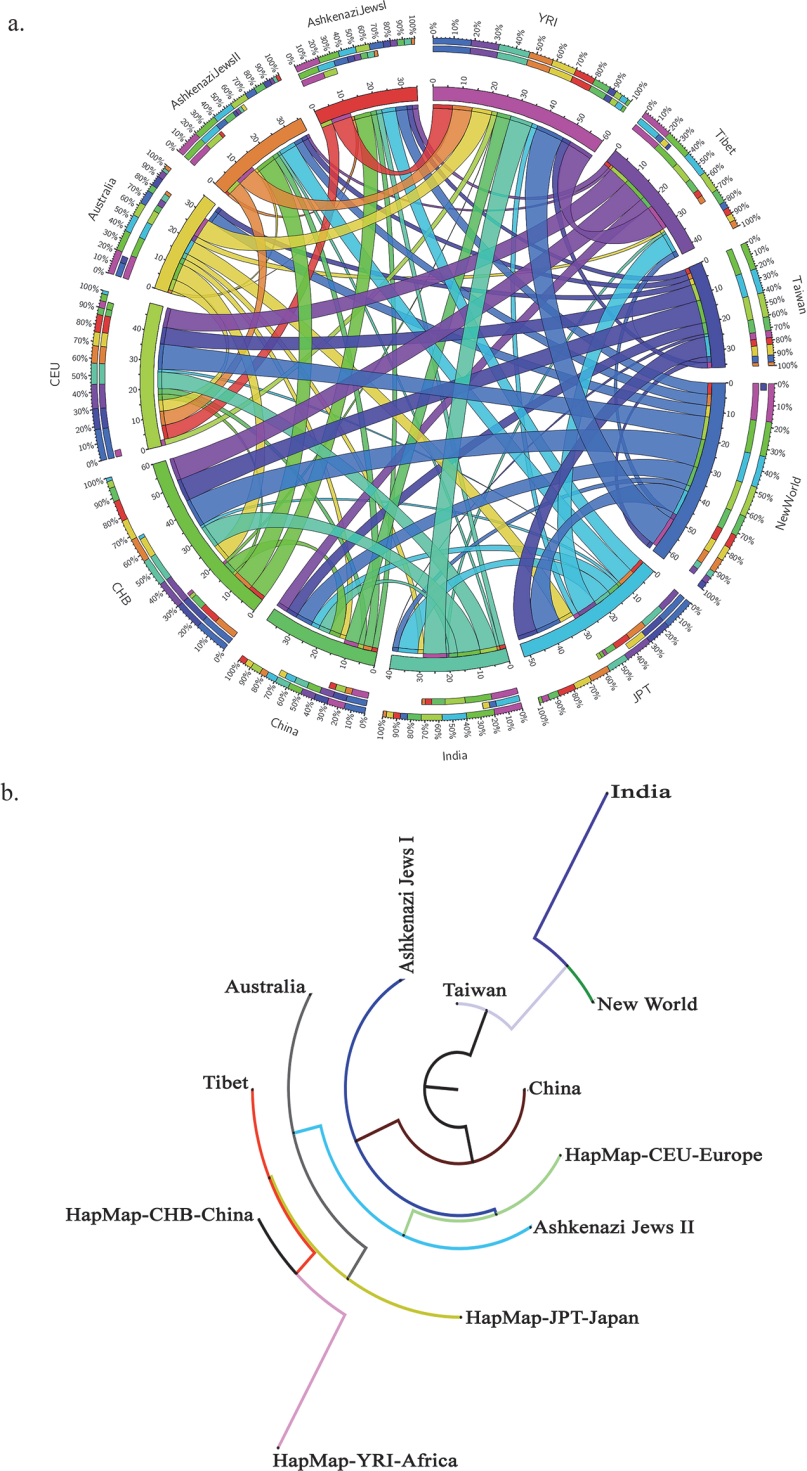
CNV map and phylogenetic tree. a) CNV map of 379 shared CNVs across all chromosomes and populations**.** The outermost to innermost tracks are: pair-wise clustering of shared CNVs in percent showing a total of >50 Mb regions across all chromosomes in this Circos image. b) A phylogenetic tree based on the CNVs in the genome. Nei's distance between any two populations was calculated based on the CN frequencies of 379 CNV regions. Phylogenetic tree was constructed based on the neighbor-joining method; Taiwan and China are closer to each other compared to the other populations.

We identified a total of 379 singleton CNV loci breakpoints from 19,905 CNVs conserved across populations (Table 2 and Fig 3A and 3B) with varying frequency of 0.01–55.5%. The usage of singleton refers to a single CNV event of a type that sometimes comes in pairs or groups. These CNV breakpoints were identified starting from the highly conserved CNVs across 12 populations upto 6 populations (S1 Table). A total of 14 breakpoints were found across all 12 populations and were found distributed in chromosomes 1–4, 8, 14, 15, 17 and 21. The highest frequency was observed for the CNV breakpoint 39235591 bp in chromosome 8p11, across all populations, and the global frequency was observed at 39.35%. The highest frequency for this breakpoint was observed in CEU (>55.55%) and the least was observed for YRI (11%). The frequency for this CNV breakpoint was high in European ethnicity, moderate in south East Asia, low in north Asia, least in YRI, but found to be highest in the New World populations. The number of conserved CNVs slightly increased for the 11 populations and this pattern remained same with the elimination of one population at a time. Similarly, 15 CNV breakpoints were seen conserved in 11 populations, followed by 21 CNVs in 10 populations, 30 CNVs in 9 populations, 82 in 8 populations, 91 in 7 populations and 133 CNVs in 6 populations. Interestingly, these conserved CNV breakpoints were identified across all chromosomes, but not in the 20^th^ chromosome.

**Table 2:** Number of shared CNV breakpoints across the study populations.

Populations	HapMap-YRI-Africa	HapMap-CEU-Europe	Ashkenazi JewsI & II	China & CHB	Tibet	India	HapMap-JPT-Japan	Australia	New World
**HapMap-YRI-Africa**	-								
**HapMap-CEU-Europe**	127	-							
**Ashkenazi Jews I & II**	229	257	-						
**China & CHB**	184	190	439	-					
**Tibet**	73	42	354	217	-				
**India**	83	86	297	186	263	-			
**HapMap-JPT-Japan**	92	72	165	170	62	61	-		
**Australia**	85	99	NA	195	67	99	67	-	
**New World**	73	64	333	219	NA	53	53	118	-
**Taiwan**	157	194	NA	468	296	149	64	284	347

NA indicates = Not Available

- indicates = Nil

### Breakpoint validations

Successful amplification of four recurring CNV breakpoints was performed on 500 randomly chosen individuals from India validating the presence of the CNVs. Varying frequency and amplification status was observed for breakpoints. About 48–54% individuals showed the presence of these CNV breakpoints at varying frequency in the selected Indian samples.

## Discussion

Identification of CNVs across diverse populations helps to understand the organization, distribution pattern of CNVs, evolutionary dynamics of the human genome, and accounts for differences in the expression of genes [27]. There have been increasing number of CNV studies using different ethnic backgrounds; however, there have not been many, which comparatively include populations across all continents to study notable variations. Here we present a comprehensive global CNV spectrum across 12 populations.

### CNV distribution

Genome-wide distribution of CNVs revealed the presence of both duplication and deletion CNV events at a varying frequency, with the New World populations showing 4-fold higher CNVs compared to Old World populations. The observed higher fold was due to the increase in only the number of duplication events, since the number of deletion events remained stable across populations. Previous studies have shown duplication CNVs to be metabolically beneficial with lower body weight by protecting from diet-induced obesity and metabolic syndrome; while deletion CNVs have shown increased body weight and metabolic syndrome-like phenotypes [28]. These were not attributed to any single gene, but rather on the CNV type distribution. All the HapMap populations except for YRI showed higher deletion CNVs. We assume that the genome can sustain more duplication than deletion as deletions have reached the threshold level. Therefore, the evidence for purifying selection against deletions seems highly plausible and those deletions may be important in the etiology of human fitness and survival.

Frequency of the CNV count across asian population was inconsistent, with only India and Tibet showing similar CNV number load, but also resembled Ashkenazi Jews. We detected gradual increase in the CNV count from Old World towards New World populations indicating selective pressure of CNV occurrences in New World populations contributing to increased genetic diversity. A majority of the discovered CNVs were found within the 100-500kb size and the remaining events gradually declined with increase in size. We observed only 13 CNV events near the 4–5 Mb range which were found localized near the heterochromatic gene poor regions, further, we did not detect any CNVs >5.5Mb. This size limitation is probably to avoid larger sized CNVs attaining fixation near the gene rich regions, which would drastically influence the gene expression and regulation. Previous studies have identified that any two individual human genomes may differ by 0.46Mb [6], ~20Mb [5], 1.06kb–1.73Mb [12], 18Mb [29] and 0.37Mb [30]. Contrary to these, a mean size of 7±3Mb was observed globally with individuals from New world populations showing the highest number of CNV size compared to moderate CNV size in South Asian populations and the lowest for HapMap populations. These CNV sizes were distinguishably limited within their geographical boundaries.

### CN state

Evaluation of the nature of zygosity for all the identified CNVs was necessary to accurately assess the genome changes, which otherwise would be improper to discuss just about duplications and deletions without correlating with its exact CN state. China, JPT, and CEU show adequate loss of genome with high 0 and 1 CN states. Australia, Tibet, India and Ashkenazi Jews show less genome loss, besides show a significant gain in the genome weight with abundant accumulation of duplications (3 and 4 CN states). Africa, considered to be phylogenetically old population was found to be stable across all CN states (except CN = 4 state) and all the remaining populations deviated in all CN states considerably. Africa showed the highest CN state of 4 across all populations indicating the founder state of the genome, which was not observed in any genome of the other populations. Therefore, we believe that the CN states are an indicator of the populations losing parts of the genome when compared to Africa. India, Tibet, Australia, Taiwan, Ashkenazi Jews and New World showed almost negligible homozygous loss of genome, instead had a significant gain in the 3 CN state. New World, China, Ashkenazi Jews and Taiwan showed higher burden of CNVs on sex chromosomes compared to CEU and India indicating the CNV influence on sex chromosomes is independent of the CNV influence conferred on autosomes. Inclusive analyses of all the 5 CN states determined the gross loss or gain of a genome in different populations, indicating the role of independent evolutionary dynamics of the genomes, and thus is an essential tool to study the evolutionary drift of the genomes in populations.

### Recurring and Rare CNVs

Of all the CNV types, moderately recurring CNVs were found more frequently in all the populations. The highest number of recurring CNVs, moderately recurring CNVs and rare CNVs were found in Africa, New World and China respectively. New World showed more of highly conserved CNVs and least number of rare CNVs whereas China showed the maximum number of rare CNVs. Several CNVs were identified as novel in some parts of the genome, which showed no prior record from previously reported CNV studies in the Database of Genomic Variations (DGV) (http://dgv.tcag.ca/dgv/app/home) [31]. Duplications were abundant in novel regions than deletions, however, no distinct pattern was observed in the distribution of novel CNV load in between New World and Old World populations.

### Minor Allele Frequency of CNV Breakpoints

We identified a total of 44,109 CNVs from 12 populations which was comparable against 1,09,863 CNVs (as of 2013) from 55 studies deposited in the Database of Genomic Variations which hence validates the ability of our arrays to detect reliable variations. Of the 44,109 CNVs identified in the current study, 19,905 CNVs were found to be ancient in origin. These conserved CNVs comprised of 379 singleton events and are identified across all populations, at varying frequencies. These CNVs across multiple ethnic populations suggest that they are evolutionarily ancient, having occurred prior to the separation of the ethnic groups and are not independently recurring events since they are identified across all populations at convincing frequency. Interestingly, 20^th^ chromosome did not contain any of the conserved CNV breakpoints. Chromosome 20 in humans, mouse, and rat are believed to be almost perfectly aligned [32–34]. This observation could be interpreted as strong evidence that this form was ancestral to mammals [35]. Contrary to this view, other reports also suggest that 20^th^ chromosome in human and mouse forms are derivative. Since, we did not find any single factor that might explain the absence of ancestral CNVs, it can be surmised that the ancestral CNVs might have been lost over time due to high rate of multiplicity.

### Path of Human Migration based on CNVs

The nonrecombining region of the Y chromosome and the sequence of mtDNA have conserved haplotype information over time and since the nucleotide substitutions had low mutation rates making them amenable for gene flow and human migration studies for more than two decades [36–41]. On the contrary, recent studies have shown elevated levels of heteroplasmy in both mtDNA and Y-chromosome at various positions, suggesting that ongoing occurrence of somatic back-mutations are unstable and intractable for human migration study [42–43]. While haplogroup calling in mtDNA sequence determines female lineage, Y-chromosome haplogroups determine male lineages. This type of haplogroup-calling results in inaccurate calling since it is shown to alter the calling through the back-mutation and therefore needs to be supplemented with other types of analyses [42].

On the other hand, CNVs represent an excellent tool to study human migration since evolutionarily ancient CNVs (from the founders) are fixed in the populations enabling the tracking of both male and female lineages. Though CNV events show high mutation rate unlike SNPs and Y chromosomal variations, however, this is circumvented by carefully avoiding the selection of random CNV events in the present study since they show back-mutation through recombination and selecting only the evolutionarily conserved CNVs. In the current study we utilized the 379 singleton CNVs from a load of 19905 CNVs along with the remaining 24204 CNVs to establish a probable path of human origin and migration. These 44109 CNV events were verified against the CNVs of every other population to identify the number and frequency of shared CNVs (S1 Table). This list of evolutionarily ancient CNVs along with the recent CNVs represents excellent candidates to help charter the path of human migration. These provide clues about the genetic relationships and diversity between and within populations. Most evidences indicate the evolution of humans began in Africa commonly known by the *displacement theory*, as "out of Africa," contend that modern human populations are derived from a single modern population group that left Africa about 60,000–80,000 years ago. This founding group is believed to have migrated throughout the Old World [44–45].

**Fig 4:**
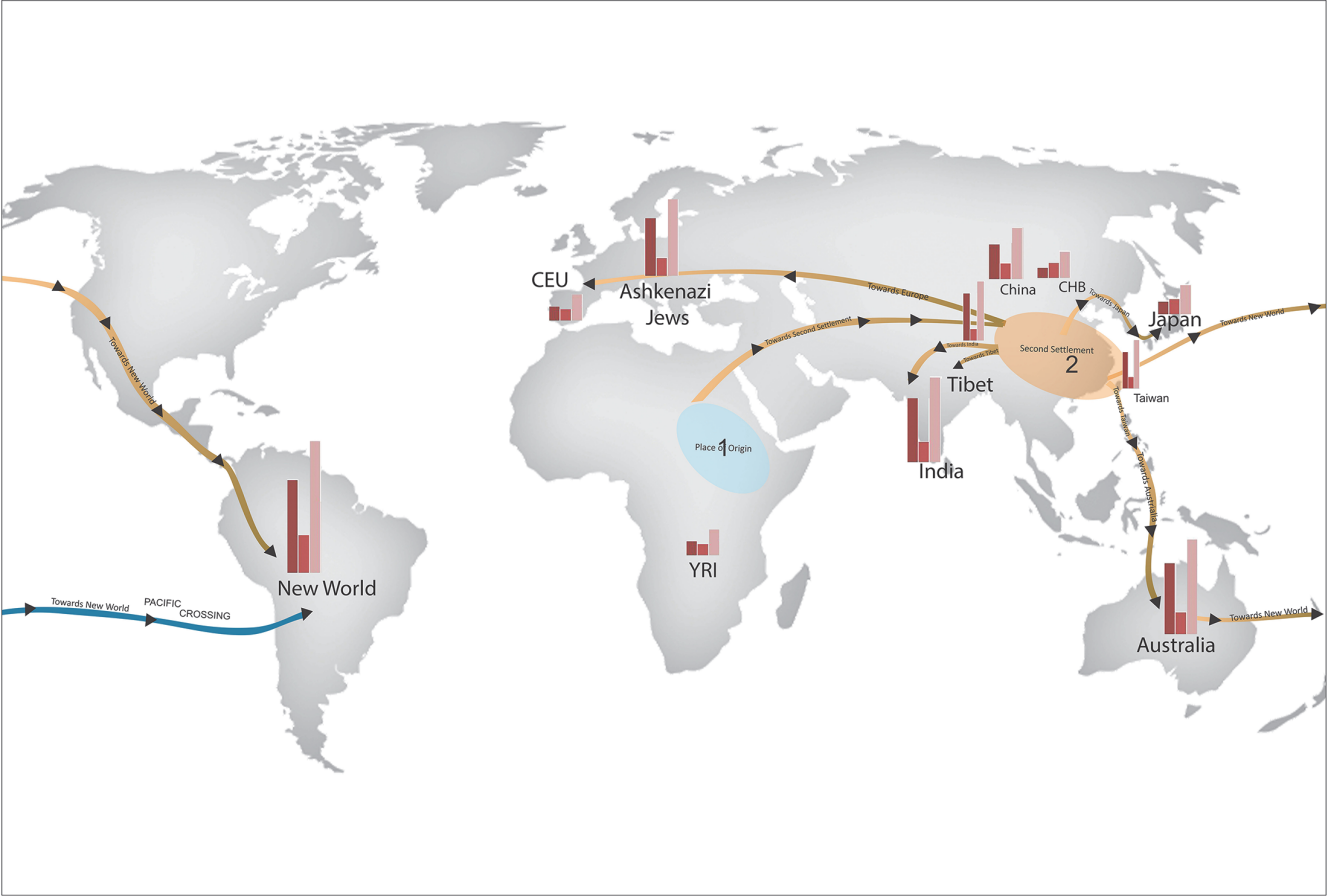
A global map showing the CNV counts of populations taken in the study and the possible migration routes. Anatomically modern humans evolved from Africa formed the first settlement which branched further towards the second settlement (SS) forming the present day east China and Taiwan regions. This SS showed increased number of shared genomic signatures with the further branched populations such as Tibet, India, Japan, Taiwan, and Australia towards the east, while Ashkenazi Jews and CEU towards the west. More than 5 branches emerged from the SS at varying time points and with varying founding members, namely SS to Tibet, SS to India, SS to Europe, SS to Japan, and SS to Taiwan. Bar graphs indicate genome-wide distribution of duplication CNVs (1st Bar), deletion CNVs (2^nd^ Bar) and CNV (3rd Bar) consists of both duplication and deletion events across 12 populations.

We propose a new human migration path based on the CNV genetic map from this study (Fig 4). Our study indicates that anatomically modern humans evolved from Africa are the most genetically diverse; some of the members of this first settlement formed a branch of *Homo sapiens* which left Africa and moved further towards the second settlement (SS) which in the present day geographical boundaries make up east China and Taiwan regions. The SS ended up with an overall number of 184 singleton CNVs, a subset of which existed in the founder population. This SS or second founding group showed increased number of shared genomic signatures with the further branched populations which make up populations such as Tibet, India, Japan, Taiwan, and Australia towards the east, while Ashkenazi Jews and CEU towards the west. We believe >5 branches to have emerged from the SS at varying time points and with varying founding members, they are, SS to Tibet, SS to India, SS to Europe, SS to Japan, and SS to Taiwan. These groups share >2–5 fold higher degree of genomic signatures with the SS, while only <1-fold with Africa (first settlement). The migration further moved downwards to reach Australia, which share 3-fold higher CNV signatures with the SS, whereas, lesser than a fold with Africa. The degrees of separation were comparatively lesser when the branched populations were compared against the SS than with the first settlement (Africa). New World, furthermore, showed >2-fold shared CNV events with both Australia and Taiwan compared to Africa. These two routes have earlier been proposed [44] and the number of shared events between New World, Australia and Taiwan correlates with their branching subset, which existed in the second founder population (Fig 4).

One of the possibilities for this event to occur could be an increase in positive selection of CNVs that might conceivably occur in populations that have migrated into new environments. Furthermore, it can been hypothesized that non-African populations have experienced more recent strong local adaptation as modern humans migrated out of Africa into novel and diverse environments. These above parameters were taken into account while recreating the migration map using CNV data.

Like any other study, this study also has limitations. Since, the proposed migration route in this study is based on CNV markers, and is from 1715 individuals belonging to 12 populations across the globe, considering the vast geographic scales and since we have unattempted to stress on individual linguistic and ethnic backgrounds of the samples, it forms a possible limitation of the study. However, the present study has generated reasonable amount of data to arrive at a conclusion for the use of CNVs as a tool to study human migration. Therefore, large scale studies in these directions will add on to our understanding on human migration.

This comprehensive whole-genome study identified a total of 44109 CNVs from 1715 genomes with a mean size of 7±3Mb which has significantly expanded our knowledge of CNVs. Duplication CNVs were significantly higher than deletion CNVs and a major portion of CNVs were within 500kb and CNV count declined with increase in size. We demonstrated the use of CNV tool to study human migration for the first time and proposed new migration routes in addition to the existing ones. The CNV map we generated provides a rationale for prioritizing population specific genome organization and uncovered the elements of the genome that had largely been unexplored. We have addressed the burden of CNVs on the coding genome more extensively in another manuscript [46].

## Supporting Information

S1 Table The population-wise and global frequency of CNVs shared across 12 populations. (DOC)
