# Supplementary material for: Global Spectrum of Copy Number Variations Reveals Genome Organizational Plasticity and Proposes New Migration Routes
Source: PLoS One. 2015 Apr 24;10(4):e0121846. doi: 10.1371/journal.pone.0121846 (PMC4409114; doi:10.1371/journal.pone.0121846)
Supplement: S1 Table — (DOC) [file pone.0121846.s001.doc]

| Table_S1. The population-wise and global frequency of CNVs shared across 12 populations. |  |  |  |  | **CNV frequency** | | | | | | | | | | | | |  |
| --- | --- | --- | --- | --- | --- | --- | --- | --- | --- | --- | --- | --- | --- | --- | --- | --- | --- | --- |
|  | Sl. No. | Chromo-  some | Cytoband | Breakpoint | HapMap  -YRI-Africa | HapMap-CEU-  Europe | Ashkenazi Jews I | Ashkenazi Jews II | HapMap  -CHB-China | China | Tibet | India | HapMap-JPT-Japan | Australia | New World | Taiwan | Global | Sum |
| **12 populations** | 1 | 1 | p36.33 | 61723 | 6.66 | 3.33 | 5.38 | 6.45 | 18.06 | 4.54 | 16.12 | 31.57 | 4.44 | 7.54 | 17.07 | 16.3 | 9.03 | 137.46 |
|  | 2 | 1 | q21.2 | 149036512 | 14.44 | 22.22 | 9.26 | 7.91 | 7.09 | 18.18 | 3.22 | 19.3 | 11.11 | 9.43 | 14.6 | 7.06 | 9.85 | 143.82 |
|  | 3 | 2 | p11.2 | 89133112 | 16.66 | 37.77 | 18.96 | 17.5 | 42.58 | 13.63 | 6.45 | 71.05 | 17.77 | 22.64 | 41.46 | 25.54 | 23.67 | 332.01 |
|  | 4 | 3 | q26.1 | 162513080 | 8.88 | 4.44 | 3.44 | 3.54 | 4.51 | 2.27 | 6.45 | 2.63 | 6.66 | 7.54 | 4.87 | 3.26 | 4.13 | 58.49 |
|  | 5 | 4 | q13.2 | 69374929 | 11.11 | 21.11 | 18.96 | 14.58 | 9.03 | 2.27 | 9.67 | 7.89 | 13.33 | 16.98 | 4.87 | 19.02 | 15.16 | 148.82 |
|  | 6 | 4 | q13.2 | 69375336 | 3.33 | 7.77 | 6.46 | 9.58 | 36.12 | 54.54 | 25.8 | 21.05 | 44.44 | 3.77 | 2.43 | 21.19 | 14.27 | 236.48 |
|  | 7 | 8 | p23.1 | 7214599 | 8.88 | 2.22 | 8.4 | 6.87 | 3.87 | 4.54 | 3.22 | 21.05 | 4.44 | 13.2 | 7.31 | 8.69 | 7.4 | 92.69 |
|  | 8 | 8 | p11.22 | 39235591 | 11.11 | 55.55 | 48.2 | 46.04 | 19.3 | 15.9 | 16.12 | 28.94 | 24.44 | 41.5 | 24.39 | 40.2 | 39.35 | 371.69 |
|  | 9 | 14 | q32.33 | 106194465 | 3.33 | 4.44 | 2.58 | 2.08 | 14.86 | 4.54 | 3.22 | 31.57 | 4.44 | 1.88 | 31.7 | 20.1 | 6.99 | 124.74 |
|  | 10 | 14 | q32.33 | 106218573 | 13.33 | 15.55 | 27.32 | 25.62 | 21.93 | 11.36 | 6.45 | 15.78 | 8.88 | 35.8 | 4.87 | 22.82 | 22.74 | 209.71 |
|  | 11 | 15 | q11.1 | 20395517 | 3.33 | 2.22 | 7.32 | 3.54 | 9.03 | 2.27 | 6.45 | 10.52 | 2.22 | 1.88 | 12.1 | 6.52 | 5.59 | 67.4 |
|  | 12 | 15 | q11.2 | 22317500 | 1.11 | 3.33 | 3.23 | 3.33 | 1.29 | 6.81 | 9.67 | 2.63 | 2.22 | 1.88 | 7.31 | 3.26 | 3.2 | 46.07 |
|  | 13 | 17 | q21.31 | 44401055 | 1.11 | 3.33 | 15.73 | 18.125 | 11.61 | 4.54 | 22.5 | 31.57 | 6.66 | 7.54 | 14.63 | 16.3 | 14.34 | 153.645 |
|  | 14 | 21 | p11.2 | 10736859 | 2.22 | 7.77 | 14.65 | 17.5 | 16.77 | 6.81 | 25.8 | 18.42 | 11.11 | 1.88 | 75.6 | 26.08 | 16.9 | 224.61 |
| **11 populations** | 15 | 3 | q26.1 | 162513137 | 15.55 | 7.77 | 7.97 | 8.75 | 21.29 | 45.45 | 16.12 | - | 44.44 | 9.43 | 7.31 | 15.21 | 12.47 | 199.29 |
|  | 16 | 4 | q13.2 | 69371991 | 38.88 | 21.11 | 5.81 | 3.54 | 1.29 | 4.54 | - | 6.45 | 2.22 | 1.88 | 2.43 | 2.17 | 6.47 | 90.32 |
|  | 17 | 4 | q13.2 | 70127632 | 14.44 | 4.44 | 8.62 | 5.41 | 2.58 | 2.27 | - | 2.63 | 13.33 | 7.54 | 12.19 | 1.63 | 6.23 | 75.08 |
|  | 18 | 8 | p23.1 | 7250368 | 10 | 10 | 3.01 | 4.16 | 7.74 | 13.63 | 12.9 | 2.03 | 4.44 | 5.66 | - | 3.8 | 5.07 | 77.37 |
|  | 19 | 8 | p23.1 | 12260915 | 1.11 | 3.33 | 3.23 | 3.125 | 1.29 | 2.27 | 3.22 | - | 2.22 | 13.2 | 9.75 | 7.06 | 3.67 | 49.805 |
|  | 20 | 8 | p23.1 | 7798839 | 1.11 | 2.22 | 0.431 | 1.45 | 0.64 | 2.27 | 3.22 | - | 2.22 | 1.88 | 7.31 | 1.63 | 1.34 | 24.381 |
|  | 21 | 14 | q11.2 | 19562127 | 4.44 | 6.66 | 6.68 | 3.75 | 3.22 | 2.27 | - | 10.52 | 2.22 | 9.43 | 4.87 | 5.97 | 5.13 | 60.03 |
|  | 22 | 14 | q32.33 | 106574548 | 1.11 | 1.11 | 1.29 | 1.25 | 1.29 | - | 6.45 | 18.42 | 2.22 | 1.88 | 2.43 | 5.97 | 2.27 | 43.42 |
|  | 23 | 15 | q11.1 | 20216943 | 6.66 | 4.44 | 1.724 | 1.875 | 1.29 | 4.54 | - | 6.45 | 6.66 | 1.88 | 2.43 | 2.71 | 2.5 | 40.659 |
|  | 24 | 15 | q11.2 | 22301994 | 5.55 | 3.33 | 3.01 | 4.16 | 10.3 | 9.09 | 6.45 | 2.63 | 8.88 | - | 7.31 | 2.17 | 4.43 | 62.88 |
|  | 25 | 15 | q11.1 | 22588019 | 3.33 | 4.44 | 9.48 | 11.45 | 11.61 | 4.54 | 6.45 | 10.52 | 4.44 | 9.43 | - | 12.5 | 9.44 | 88.19 |
|  | 26 | 17 | q21.31 | 44394400 | 10 | 20 | 9.69 | 10.62 | 21.93 | 2.5 | 29.03 | 18.52 | 33.33 | - | 26.82 | 16.3 | 13.99 | 198.74 |
|  | 27 | 19 | q13.2 | 43294378 | 5.55 | 3.33 | 0.64 | 0.2 | 7.09 | 2.27 | 6.45 | - | 8.88 | 3.77 | 2.43 | 2.71 | 2.21 | 43.32 |
|  | 28 | 22 | q11.23 | 24396802 | 1.11 | - | 0.21 | 0.83 | 0.64 | 2.27 | 3.22 | 2.63 | 2.22 | 1.88 | 2.43 | 1.08 | 0.87 | 18.52 |
|  | 29 | 22 | q11.1 | 16055171 | 11.11 | 8.88 | 20.47 | 21.87 | 4.51 | - | 12.9 | 28.9 | 6.66 | 22.64 | 9.75 | 21.19 | 17.37 | 168.88 |
| **10 populations** | 30 | Y | p11.2 | 3826212 | 3.33 | 8.88 | 13.14 | 11.87 | 11.61 | 6.81 | 25.8 | 13.15 | 4.44 | 5.66 | - | 14.13 | 11.31 | 118.82 |
|  | 31 | 1 | p36.13 | 17011438 | 2.22 | 2.22 | 1.07 | 0.41 | 3.22 | 4.54 | - | 2.63 | - | 1.88 | 2.43 | 1.63 | 1.39 | 22.25 |
|  | 32 | 2 | p11.2 | 89136040 | 1.11 | - | 28.23 | 38.54 | 18.7 | 2.27 | 6.45 | 21.05 | 2.22 | 32.07 | 26.82 | 42.39 | 27.05 | 219.85 |
|  | 33 | 2 | p11.2 | 89934947 | 1.11 | 4.44 | 0.64 | 1.25 | 3.22 | 4.54 | 3.22 | - | 4.44 | - | 12.19 | 3.8 | 2.09 | 38.85 |
|  | 34 | 2 | p11.2 | 89995025 | 3.33 | - | 2.37 | 0.01 | 1.29 | 2.27 | - | 2.63 | 4.44 | 11.3 | 4.87 | 4.89 | 2.68 | 37.4 |
|  | 35 | 3 | q26.1 | 162513447 | 2.22 | - | 0.21 | 0.41 | 5.16 | 4.54 | 9.67 | 2.63 | 6.66 | - | 2.43 | 1.63 | 1.51 | 35.56 |
|  | 36 | 5 | q13.2 | 68846291 | 7.77 | - | 1.93 | 3.54 | 0.64 | 2.27 | 3.22 | 18.42 | - | 15.09 | 4.87 | 5.97 | 3.73 | 63.72 |
|  | 37 | 7 | q35 | 143917589 | 1.11 | 2.22 | 8.18 | 6.45 | 2.58 | 2.27 | 3.22 | 21.05 | - | - | 12.19 | 10.86 | 6.47 | 70.13 |
|  | 38 | 14 | q11.1 | 19002112 | 2.22 | 1.11 | 7.97 | 4.16 | 6.45 | - | 3.22 | 2.63 | - | 7.54 | 26.82 | 9.23 | 6.06 | 71.35 |
|  | 39 | 14 | q11.2 | 20200171 | 2.22 | 5.55 | 0.64 | 1.66 | 1.93 | 6.81 | - | 5.26 | 8.88 | 1.88 | - | 1.08 | 1.92 | 35.91 |
|  | 40 | 14 | q32.33 | 106547302 | 1.11 | 4.44 | 6.68 | 7.08 | 2.58 | - | 6.45 | 2.63 | - | 1.88 | 7.31 | 5.43 | 5.3 | 45.59 |
|  | 41 | 15 | q14 | 34695310 | 1.11 | 1.11 | 0.21 | 0.62 | 2.58 | 79.09 | - | 5.26 | 2.22 | - | 7.31 | 1.63 | 1.34 | 101.14 |
|  | 42 | 15 | q11.1 | 22681064 | - | 1.11 | 4.52 | 8.12 | 0.64 | - | 6.45 | 5.26 | 4.44 | 5.66 | 19.51 | 8.69 | 4.95 | 64.4 |
|  | 43 | 16 | p12.2 | 22558299 | - | 4.44 | 2.15 | 1.45 | 6.45 | 4.54 | 3.22 | 2.63 | 11.11 | - | 2.43 | 3.26 | 2.74 | 41.68 |
|  | 44 | 16 | p11.2 | 32280344 | 1.11 | 1.11 | 0.21 | 0.62 | 1.29 | 4.54 | - | 2.63 | 4.44 | 1.88 | - | 2.17 | 1.04 | 20 |
|  | 46 | 19 | p12 | 20716377 | 3.33 | 1.11 | 8.4 | 5.62 | 5.16 | 2.27 | - | 5.26 | 2.22 | 3.77 | - | 2.17 | 5.13 | 39.31 |
|  | 47 | 22 | q11.22 | 23223233 | 6.66 | 21.11 | 9.91 | 4.79 | 9.67 | 9.09 | - | 7.89 | 26.66 | 3.77 | - | 9.23 | 8.39 | 108.78 |
|  | 48 | X | q21.31 | 91672378 | 2.22 | 5.55 | 1.077 | 1.875 | 5.16 | - | - | 5.26 | 11.11 | 5.66 | 9.75 | 6.52 | 3.2 | 54.182 |
|  | 49 | Y | p11.2 | 3439401 | 2.22 | 4.44 | 2.37 | 0.625 | 1.29 | 2.27 | 3.22 | - | 6.66 | 5.66 | - | 1.63 | 1.98 | 30.385 |
| **9 Populations** | 50 | 1 | q21.2 | 148530424 | - | 1.11 | 0.21 | 0.41 | 1.93 | 4.54 | 3.22 | - | 4.44 | - | 2.43 | 0.54 | 0.81 | 18.83 |
|  | 51 | 1 | q21.2 | 148916177 | - | 3.33 | 1.72 | 0.83 | 1.29 | 2.27 | - | 2.63 | 2.22 | - | 2.43 | 2.17 | 1.45 | 18.89 |
|  | 52 | 1 | p21.1 | 104066865 | 1.11 | 3.33 | 1.07 | 1.87 | 3.87 | - | 6.45 | - | 2.22 | - | 2.43 | 0.54 | 1.69 | 22.89 |
|  | 53 | 2 | p11.2 | 90267091 | 5.55 | 3.33 | 1.07 | 0.62 | 1.29 | 2.27 | - | 2.63 | 4.44 | - | - | 4.89 | 1.8 | 26.09 |
|  | 54 | 4 | q35.2 | 191020138 | - | - | 4.31 | 2.7 | 7.09 | - | 25.8 | 18.42 | 2.22 | 30.18 | 31.7 | 22.28 | 7.58 | 144.7 |
|  | 55 | 5 | q13.2 | 68867282 | 7.77 | 5.55 | 10.77 | 9.37 | 7.09 | - | 3.22 | - | - | 9.43 | 4.87 | 5.97 | 7.98 | 64.04 |
|  | 56 | 6 | p25.3 | 257341 | 1.11 | 3.33 | 4.31 | 4.58 | 5.16 | 9.09 | 3.22 | - | 15.55 | - | - | 5.97 | 4.48 | 52.32 |
|  | 57 | 8 | p23.1 | 11987714 | 1.11 | 1.11 | 7.97 | 8.33 | 1.93 | - | 3.22 | - | - | 9.43 | 7.31 | 8.69 | 6.23 | 49.1 |
|  | 58 | 9 | p12 | 41892854 | - | 2.22 | 2.58 | 2.7 | 2.58 | - | 3.22 | 5.26 | 2.22 | 5.66 | - | 2.71 | 2.5 | 29.15 |
|  | 59 | 9 | p12 | 43505840 | 2.22 | 5.55 | 5.12 | 5.41 | 4.51 | - | 3.22 | 7.89 | - | 5.66 | - | 4.89 | 4.66 | 44.47 |
|  | 60 | 9 | p11.2 | 43612150 | 3.33 | - | 1.72 | 1.25 | 3.22 | 2.27 | - | 7.89 | 2.22 | - | 2.43 | 2.17 | 1.86 | 26.5 |
|  | 61 | 9 | p11.2 | 44727847 | 2.22 | 4.44 | 11.42 | 12.5 | 10.32 | - | 9.67 | 7.89 | - | - | 21.95 | 9.23 | 9.73 | 89.64 |
|  | 62 | 10 | q11.22 | 47743504 | 2.22 | 2.22 | 2.15 | 3.12 | 2.58 | - | 3.22 | 2.63 | - | - | 2.43 | 3.26 | 2.44 | 23.83 |
|  | 63 | 12 | p13.31 | 8004413 | - | 4.44 | 3.23 | 1.66 | 1.93 | 2.27 | 6.45 | - | 4.44 | 1.88 | - | 4.34 | 2.56 | 30.64 |
|  | 64 | 15 | q11.2 | 22280465 | 7.77 | 8.88 | 5.38 | 3.54 | 5.8 | 2.27 | - | 2.63 | - | 1.88 | - | 3.8 | 4.43 | 41.95 |
|  | 65 | 16 | p13.11 | 14989851 | - | - | 1.07 | 1.04 | 3.22 | 2.27 | 3.22 | - | 2.22 | 1.88 | 2.43 | 2.17 | 1.39 | 19.52 |
|  | 66 | 16 | p11.2 | 32380939 | 3.33 | 2.22 | 3.66 | 2.29 | 3.87 | 9.09 | - | - | 2.22 | 3.77 | - | 2.71 | 2.97 | 33.16 |
|  | 67 | 16 | p11.2 | 33404128 | 1.11 | 5.55 | 1.07 | 1.04 | 1.29 | 9.09 | - | - | 2.22 | 1.88 | - | 1.63 | 1.57 | 24.88 |
|  | 68 | 16 | p11.2 | 32058483 | 3.33 | 5.55 | 1.29 | 1.87 | 3.22 | 9.09 | - | - | 6.66 | - | 2.43 | 3.26 | 2.44 | 36.7 |
|  | 69 | 16 | p11.2 | 33380754 | - | 3.33 | 9.63 | 10.41 | 5.16 | 2.27 | 3.22 | - | 4.44 | - | 4.87 | 4.34 | 6.99 | 47.67 |
|  | 70 | 17 | p11.2 | 18355380 | 1.11 | 4.44 | 9.26 | 8.12 | 2.58 | - | - | 5.26 | 2.22 | 1.88 | - | 5.97 | 6.18 | 40.84 |
|  | 71 | 17 | q21.31 | 44408650 | 5.55 | - | 0.86 | 0.41 | 1.29 | 4.54 | - | 5.26 | 4.44 | - | 2.43 | 2.17 | 1.39 | 26.95 |
|  | 72 | 17 | q21.31 | 44428669 | - | 1.11 | - | 0.2 | 1.93 | 2.27 | 3.22 | - | 4.44 | 1.88 | 4.87 | 2.17 | 0.93 | 22.09 |
|  | 73 | 17 | q12 | 34629684 | 16.66 | 2.22 | 0.43 | 1.25 | 3.87 | 4.54 | 12.9 | - | 11.11 | - | - | 3.26 | 2.79 | 56.24 |
|  | 74 | 19 | p13.3 | 90898 | - | 1.11 | 1.94 | 1.04 | 4.51 | - | 3.22 | 2.63 | - | 3.77 | 7.31 | 4.34 | 2.15 | 29.87 |
|  | 75 | 19 | p12 | 20596194 | 8.88 | 5.55 | 2.58 | 1.66 | 12.25 | 18.18 | - | 15.78 | 24.44 | - | - | 5.43 | 5.07 | 94.75 |
|  | 76 | 22 | q11.22 | 23040138 | 1.11 | - | 3.66 | 0.41 | 1.29 | - | 6.45 | 2.63 | 2.22 | 1.88 | - | 2.17 | 1.8 | 21.82 |
|  | 77 | 22 | q11.22 | 23090867 | 5.55 | - | 3.01 | 3.54 | 0.64 | 2.27 | 6.45 | 2.63 | - | - | 2.43 | 1.08 | 2.56 | 27.6 |
|  | 78 | Y | p11.2 | 4670883 | - | - | 5.6 | 5 | 9.67 | 2.27 | 25.8 | 7.89 | 4.44 | 3.77 | - | 3.8 | 5.13 | 68.24 |
|  | 79 | Y | p11.2 | 5683812 | 1.11 | - | 1.29 | 1.45 | 7.09 | 2.27 | 9.67 | 2.63 | 6.66 | - | - | 0.54 | 1.98 | 32.71 |
|  | 80 | 1 | p36.21 | 12882985 | - | - | 3.87 | 4.79 | 1.29 | - | 9.67 | 10.52 | - | 1.88 | 2.43 | 0.54 | 3.09 | 34.99 |
|  | 81 | 1 | p36.13 | 17029580 | - | - | 1.72 | 3.95 | 2.58 | - | 19.35 | 2.63 | - | 1.88 | 9.75 | 3.8 | 2.91 | 45.66 |
| **8 Populations** | 82 | 1 | p36.13 | 16831863 | 2.22 | 1.11 | 0.64 | 0.41 | 2.58 | - | - | 2.63 | 2.22 | - | - | 1.08 | 0.93 | 12.89 |
|  | 83 | 1 | q21.2 | 149436830 | 1.11 | 4.44 | 0.43 | 1.04 | 0.64 | - | - | - | 2.22 | 5.66 | - | 1.63 | 1.16 | 17.17 |
|  | 84 | 1 | q23.3 | 161638530 | 2.22 | 2.22 | 0.64 | 0.41 | 0.64 | 4.54 | 3.22 | 2.63 | - | - | - | - | 0.81 | 16.52 |
|  | 85 | 1 | p36.13 | 17029580 | - | - | 1.72 | 3.95 | 2.58 | - | 19.35 | 2.63 | - | 1.88 | 9.75 | 3.8 | 2.91 | 45.66 |
|  | 86 | 2 | p11.2 | 90011126 | 3.33 | 2.22 | 0.43 | 1.45 | 1.29 | 2.27 | - | - | - | 1.88 | - | 0.08 | 1.16 | 12.95 |
|  | 87 | 2 | p11.2 | 89497676 | - | 2.22 | 0.21 | 1.04 | 4.51 | 2.27 | 16.12 | - | 2.22 | - | - | 0.54 | 1.34 | 29.13 |
|  | 88 | 2 | p11.2 | 89501226 | 1.11 | 1.11 | 1.29 | 0.83 | - | 2.27 | 3.22 | - | - | - | 2.43 | 0.54 | 0.93 | 12.8 |
|  | 89 | 4 | p11 | 49093774 | - | - | 1.5 | 4.37 | 3.22 | - | 3.22 | 2.63 | 2.22 | - | 9.75 | 3.26 | 2.68 | 30.17 |
|  | 90 | 4 | q35.2 | 190837871 | - | - | 0.43 | 0.2 | 0.64 | - | 6.45 | 2.63 | 2.22 | - | 4.87 | 3.26 | 0.93 | 20.7 |
|  | 91 | 4 | q13.2 | 70232112 | 2.22 | 2.22 | 0.64 | 0.83 | 0.64 | - | 6.45 | 2.63 | - | - | - | 0.54 | 0.93 | 16.17 |
|  | 92 | 5 | q13.2 | 69074965 | - | 2.22 | 22.84 | 9.16 | 0.64 | - | 3.22 | 10.52 | - | - | 2.43 | 4.34 | 9.73 | 55.37 |
|  | 93 | 7 | q35 | 143896240 | 8.88 | 2.22 | 6.25 | 5.2 | 0.64 | 4.54 | - | - | - | 5.66 | - | 4.34 | 4.54 | 37.73 |
|  | 94 | 7 | q35 | 143909223 | - | 1.11 | 3.87 | 3.33 | 1.29 | - | 3.22 | 10.52 | - | - | 2.43 | 4.34 | 2.97 | 30.11 |
|  | 95 | 8 | p23.1 | 7170477 | 2.22 | - | 2.8 | 2.29 | 3.87 | - | - | 5.26 | - | 1.88 | 4.87 | 2.71 | 2.44 | 25.9 |
|  | 96 | 8 | p23.1 | 7186620 | 1.11 | - | 3.66 | 1.87 | - | - | 3.22 | 2.63 | - | 5.66 | 2.43 | 2.17 | 2.15 | 22.75 |
|  | 97 | 8 | p23.1 | 11881847 | - | - | 0.21 | 0.41 | 0.64 | - | 6.45 | 5.26 | - | 1.88 | 17.07 | 11.95 | 2.21 | 43.87 |
|  | 98 | 8 | p23.1 | 12034295 | 1.11 | - | 0.86 | 1.45 | 3.22 | - | 3.22 | - | - | 1.88 | 2.43 | 1.08 | 1.28 | 15.25 |
|  | 99 | 8 | p11.22 | 39230171 | - | 2.22 | 3.66 | 3.95 | 1.29 | - | 6.45 | 2.63 | - | 15.09 | - | 2.17 | 3.2 | 37.46 |
|  | 100 | 8 | p11.22 | 39230647 | - | - | 3.87 | 5.62 | 1.93 | - | 3.22 | 7.89 | - | 7.54 | 24.39 | 7.06 | 4.6 | 61.52 |
|  | 101 | 8 | p11.22 | 39246663 | 1.11 | 1.11 | 4.09 | 3.75 | 1.29 | 2.27 | - | - | - | - | 2.43 | 0.54 | 2.56 | 16.59 |
|  | 102 | 8 | p23.1 | 7787950 | 1.11 | 2.22 | 0.21 | 0.41 | 0.64 | - | - | 2.63 | 2.22 | - | - | 1.63 | 0.69 | 11.07 |
|  | 103 | 8 | p23.1 | 12390152 | - | - | 3.44 | 2.29 | 0.64 | - | 3.22 | 5.26 | - | 3.77 | 2.43 | 0.54 | 2.04 | 21.59 |
|  | 104 | 9 | p12 | 43315962 | 3.33 | - | 1.5 | 1.04 | 1.29 | 4.54 | - | 2.63 | 6.66 | - | - | 0.54 | 1.39 | 21.53 |
|  | 105 | 9 | p11.2 | 45471393 | - | - | 6.03 | 12.29 | 0.64 | - | 29.03 | 5.26 | 2.22 | 24.52 | - | 9.78 | 7.63 | 89.77 |
|  | 106 | 9 | q21.11 | 69695988 | 6.66 | 1.11 | 0.64 | 0.62 | 0.64 | - | - | 2.63 | - | - | 2.43 | 0.54 | 0.99 | 15.27 |
|  | 107 | 9 | q13 | 67626353 | - | - | 0.64 | 0.2 | 1.29 | - | 16.12 | 2.63 | - | 1.88 | 2.43 | 2.17 | 1.04 | 27.36 |
|  | 108 | 9 | p11.2 | 47217164 | - | 1.11 | 2.37 | 3.75 | 3.22 | - | 3.22 | - | - | 5.66 | 2.43 | 2.17 | 2.56 | 23.93 |
|  | 109 | 9 | p11.2 | 45749096 | - | 1.11 | 1.07 | 1.04 | 0.64 | 2.27 | 3.22 | 10.52 | - | - | - | 2.17 | 1.28 | 22.04 |
|  | 110 | 9 | p11.2 | 44243422 | - | 7.77 | 0.86 | 0.62 | 4.51 | 4.54 | 3.22 | - | 2.22 | - | - | 1.08 | 1.57 | 24.82 |
|  | 111 | 9 | p11.2 | 44251216 | - | 6.66 | 7.75 | 11.87 | - | 4.54 | - | 2.63 | - | 7.54 | 7.31 | 12.5 | 7.69 | 60.8 |
|  | 112 | 10 | q11.22 | 47541177 | 4.44 | 1.11 | 0.43 | 0.41 | 0.64 | 4.54 | - | - | - | - | 2.43 | 0.54 | 0.81 | 14.54 |
|  | 113 | 10 | q11.22 | 48771938 | 2.22 | - | 0.64 | 1.45 | 1.93 | - | - | 2.63 | 2.22 | - | 4.87 | 2.71 | 1.39 | 18.67 |
|  | 114 | 10 | q11.22 | 48928779 | - | 1.11 | 1.29 | 0.62 | 3.22 | - | 3.22 | - | - | 9.43 | 4.87 | 2.71 | 1.63 | 26.47 |
|  | 115 | 12 | p11.1 | 34434964 | - | - | 26.72 | 27.08 | 1.29 | - | 3.22 | 2.63 | - | 22.64 | 14.63 | 10.86 | 16.67 | 109.07 |
|  | 116 | 12 | p11.1 | 34444790 | - | - | 13.36 | 12.9 | 4.51 | - | 6.45 | 2.63 | - | 1.88 | 41.46 | 15.21 | 10.49 | 98.4 |
|  | 117 | 14 | q11.2 | 19437975 | 2.22 | 1.11 | 6.25 | 6.87 | 1.93 | - | - | 2.63 | - | 3.77 | - | 2.17 | 4.37 | 26.95 |
|  | 118 | 14 | q11.2 | 19453314 | 2.22 | 1.11 | 0.64 | 2.08 | 1.93 | - | 6.45 | 2.63 | - | - | - | 1.63 | 1.45 | 18.69 |
|  | 119 | 14 | q11.2 | 20105479 | 2.22 | 6.66 | 3.87 | 6.04 | 1.93 | 4.54 | - | 2.63 | - | - | - | 1.63 | 3.73 | 29.52 |
|  | 120 | 14 | q32.33 | 106029625 | - | 4.44 | 9.91 | 7.28 | 3.87 | - | 25.8 | - | - | 13.2 | 2.43 | 11.95 | 7.52 | 78.88 |
|  | 121 | 14 | q32.33 | 106154856 | - | 1.11 | 1.72 | 0.41 | 5.8 | - | 3.22 | - | - | 1.88 | 7.31 | 1.63 | 1.63 | 23.08 |
|  | 122 | 14 | q32.33 | 106246290 | - | - | 4.09 | 5.83 | 7.74 | 2.27 | 9.67 | 13.15 | - | 3.77 | - | 2.71 | 4.37 | 49.23 |
|  | 123 | 14 | q32.33 | 106451029 | - | - | 1.29 | 2.5 | 12.25 | - | 16.12 | 5.26 | - | 1.88 | 9.75 | 4.89 | 3.38 | 53.94 |
|  | 124 | 14 | q32.33 | 106541741 | - | - | 1.07 | 0.62 | 1.29 | 2.27 | 6.45 | 10.52 | - | - | 2.43 | 1.08 | 1.16 | 25.73 |
|  | 125 | 14 | q32.33 | 106567088 | - | 2.22 | 5.38 | 6.25 | 4.51 | - | - | 28.94 | 2.22 | 7.54 | - | 6.52 | 5.36 | 63.58 |
|  | 126 | 14 | q32.33 | 106569451 | - | 2.22 | 5.81 | 3.54 | 3.87 | - | - | 2.63 | - | 7.54 | 4.87 | 2.71 | 3.73 | 33.19 |
|  | 127 | 14 | q32.33 | 106577580 | 1.11 | 3.33 | 4.09 | 3.95 | 0.64 | - | - | - | 2.22 | - | 2.43 | 2.71 | 2.91 | 20.48 |
|  | 128 | 14 | q11.1 | 20423360 | - | - | 9.48 | 6.45 | 1.29 | 4.54 | 3.22 | 2.63 | 4.44 | - | - | 2.17 | 5.07 | 34.22 |
|  | 129 | 14 | q32.33 | 106783032 | - | 1.11 | 1.07 | 0.83 | 3.87 | 2.27 | 3.22 | - | - | - | 2.43 | 1.08 | 1.22 | 15.88 |
|  | 130 | 14 | q32.33 | 106802041 | 2.22 | 1.11 | 0.21 | 0.2 | 0.64 | 2.27 | - | 2.63 | - | - | - | 1.08 | 0.58 | 10.36 |
|  | 131 | 15 | q11.1 | 20284054 | 4.44 | 2.22 | 0.21 | 0.41 | 0.64 | 4.54 | - | 2.63 | - | - | - | 1.08 | 0.87 | 16.17 |
|  | 132 | 15 | q11.1 | 20585976 | - | - | 2.15 | 2.08 | 5.8 | 2.27 | 16.12 | 21.05 | - | - | 12.19 | 16.84 | 4.6 | 78.5 |
|  | 133 | 15 | q11.2 | 24578487 | 1.11 | 2.22 | 0.43 | 0.62 | 2.58 | 6.81 | 3.22 | - | 2.22 | - | - | - | 0.99 | 19.21 |
|  | 134 | 16 | p11.2 | 32146874 | 1.11 | 1.11 | - | 0.2 | 1.29 | - | 3.22 | 2.63 | 2.22 | - | - | 1.63 | 0.64 | 13.41 |
|  | 135 | 16 | p11.2 | 32433041 | 3.33 | 2.22 | 2.15 | 1.45 | 1.29 | 6.81 | - | - | 4.44 | - | - | 1.08 | 1.8 | 22.77 |
|  | 136 | 16 | p11.2 | 32791257 | 1.11 | 1.11 | 1.07 | 0.83 | 1.29 | - | 16.12 | - | - | - | 7.31 | 3.8 | 1.63 | 32.64 |
|  | 137 | 16 | p11.2 | 33338112 | - | 1.11 | 2.37 | 3.33 | 5.16 | 4.54 | - | 7.89 | 11.11 | - | - | 2.17 | 2.91 | 37.68 |
|  | 138 | 17 | q12 | 34528113 | - | 1.11 | 0.86 | 1.04 | 3.22 | 2.27 | 9.67 | - | - | 1.88 | - | 2.71 | 1.45 | 22.76 |
|  | 139 | 17 | q21.31 | 44165803 | - | 42.22 | 11.42 | 18.33 | 1.29 | - | - | 63.15 | - | 7.54 | 2.43 | 9.78 | 13.29 | 156.16 |
|  | 140 | 17 | q21.31 | 44435442 | - | 3.33 | 1.93 | 0.83 | - | 4.54 | - | 5.26 | 2.22 | 1.88 | - | 1.63 | 1.45 | 21.62 |
|  | 141 | 17 | q21.31 | 44752288 | 1.11 | 7.77 | 4.74 | 4.16 | 0.64 | - | - | - | - | 7.54 | 2.43 | 4.34 | 3.73 | 32.73 |
|  | 142 | 22 | q11.22 | 23213354 | - | 2.22 | - | - | 3.22 | 2.27 | - | 10.52 | 2.22 | - | 7.31 | 1.08 | 1.04 | 28.84 |
|  | 143 | 22 | q11.23 | 25918709 | 4.44 | 2.22 | 0.86 | 1.45 | 1.29 | 2.27 | - | - | - | 1.88 | - | 2.71 | 1.51 | 17.12 |
|  | 144 | 22 | q11.22 | 23083891 | 1.11 | 4.44 | 6.03 | 2.91 | 1.29 | - | 3.22 | - | - | 9.43 | - | 1.63 | 3.38 | 30.06 |
|  | 145 | 22 | q11.22 | 23099160 | 1.11 | 2.22 | - | 1.04 | 0.64 | - | - | 2.63 | 2.22 | 1.88 | - | 2.17 | 0.93 | 13.91 |
|  | 146 | 22 | q11.22 | 23101771 | - | 1.11 | 0.21 | 0.62 | 0.64 | 2.27 | 3.22 | - | - | 1.88 | - | 0.54 | 0.58 | 10.49 |
|  | 147 | 22 | q11.22 | 23111958 | - | 1.11 | 6.03 | 9.16 | 1.93 | 2.27 | 12.9 | 2.63 | - | - | - | 2.17 | 5.01 | 38.2 |
|  | 148 | 22 | q11.23 | 25663973 | 3.33 | 12.22 | 0.86 | 0.41 | 1.29 | 6.81 | - | - | 2.22 | - | - | 0.54 | 1.57 | 27.68 |
|  | 149 | 15 | q11.1 | 20016316 | - | - | 2.37 | 3.95 | 9.03 | - | 9.67 | 13.15 | - | 7.54 | 17.07 | 9.78 | 4.72 | 72.56 |
|  | 150 | X | q21.31 | 88494865 | 1.11 | 1.11 | 0.21 | 0.62 | 3.22 | - | - | - | 4.44 | - | 2.43 | 1.63 | 0.99 | 14.77 |
|  | 151 | X | q21.31 | 89718718 | - | 1.11 | 6.03 | 7.29 | 7.09 | - | 3.22 | - | 2.22 | 3.77 | - | 2.17 | 4.83 | 32.9 |
|  | 152 | X | q21.31 | 89722770 | - | - | 2.58 | 3.33 | 2.58 | 2.27 | 6.45 | - | 2.22 | 9.43 | - | 1.63 | 2.56 | 30.49 |
|  | 153 | X | q21.31 | 91170663 | - | - | 18.96 | 13.54 | 3.22 | - | 19.35 | 2.63 | - | 3.77 | 7.31 | 7.06 | 10.67 | 75.84 |
|  | 154 | X | q21.31 | 91699891 | - | - | 9.91 | 8.75 | 9.67 | - | 6.45 | - | - | 1.88 | 17.07 | 3.8 | 7.05 | 57.53 |
|  | 155 | X | q21.32 | 91934281 | - | - | 7.32 | 11.04 | 7.09 | - | 3.22 | 5.26 | - | 3.77 | 9.75 | 9.23 | 7.23 | 56.68 |
|  | 156 | X | q21.31 | 92358869 | - | - | 0.64 | 1.87 | 1.93 | - | 6.45 | 2.63 | - | 3.77 | 9.75 | 11.41 | 2.62 | 38.45 |
|  | 157 | Y | p11.2 | 4341114 | - | 1.11 | 0.86 | 1.04 | 1.93 | - | 9.67 | 5.26 | 2.22 | - | - | 2.17 | 1.34 | 24.26 |
|  | 158 | Y | q11.223 | 28457325 | 1.11 | - | 0.43 | 0.2 | 1.93 | 2.27 | 3.22 | - | - | - | 2.43 | 0.54 | 0.64 | 12.13 |
|  | 159 | 1 | p36.21 | 12845851 | 1.11 | 4.44 | 0.86 | 0.41 | 0.64 | - | - | - | 2.22 | 1.88 | - | - | 0.81 | 11.56 |
|  | 160 | 1 | p36.21 | 12867078 | 1.11 | - | - | 0.41 | 1.93 | - | 3.22 | - | 2.22 | 3.77 | - | 0.54 | 0.64 | 13.2 |
|  | 161 | 1 | q21.2 | 148947698 | - | 1.11 | 0.64 | 0.62 | 1.29 | - | 6.45 | 6 | - | 1.88 | - | - | 1.04 | 17.99 |
|  | 162 | 1 | q23.3 | 161517939 | - | - | 2.8 | 2.29 | 2.58 | - | - | 2.63 | - | 5.66 | 4.87 | 1.08 | 2.09 | 21.91 |
|  | 163 | 1 | q31.3 | 196812518 | - | - | 1.72 | 2.08 | 0.64 | - | 3.22 | - | - | 5.66 | 9.75 | 4.34 | 2.04 | 27.41 |
|  | 164 | 1 | p36.21 | 12845851 | 1.11 | 4.44 | 0.86 | 0.41 | 0.64 | - | - | - | 2.22 | 1.88 | - | - | 0.81 | 11.56 |
|  | 165 | 1 | p36.21 | 12867078 | 1.11 | - | - | 0.41 | 1.93 | - | 3.22 | - | 2.22 | 3.77 | - | 0.54 | 0.64 | 13.2 |
| **7 Populations** | 166 | 1 | p36.21 | 12882985 | - | - | - | 4.79 | 1.29 | - | 9.67 | 10.52 | - | 1.88 | 2.43 | 0.54 | 3.09 | 31.12 |
|  | 167 | 2 | p11.2 | 87373881 | 2.22 | 5.55 | 1.29 | 2.29 | 0.64 | - | - | 2.63 | - | - | - | 0.54 | 1.57 | 15.16 |
|  | 168 | 2 | p11.2 | 89161253 | 9.99 | 13.33 | 0.43 | 0.2 | - | 6.81 | - | - | 8.88 | 15.09 | - | - | 2.07 | 54.73 |
|  | 169 | 2 | p11.2 | 90321385 | - | - | 2.8 | 1.45 | 1.29 | 2.27 | - | - | 6.66 | 3.77 | - | 0.54 | 1.69 | 18.78 |
|  | 170 | 2 | p11.2 | 89408642 | - | - | 6.89 | 5.62 | 0.64 | - | 12.9 | 2.63 | - | 5.66 | - | 3.26 | 4.31 | 37.6 |
|  | 171 | 3 | q26.1 | 162625983 | - | 1.11 | 2.8 | 1.87 | 1.29 | - | - | 2.63 | - | - | 2.43 | 0.54 | 1.63 | 12.67 |
|  | 172 | 4 | p11 | 49148822 | - | 1.11 | 3.23 | 7.29 | 0.64 | - | 3.22 | - | - | 1.88 | - | 4.89 | 3.67 | 22.26 |
|  | 173 | 4 | q13.2 | 70128400 | 13.33 | 1.11 | - | 0.62 | 0.64 | 2.27 | - | - | - | 1.88 | - | 1.08 | 1.22 | 20.93 |
|  | 174 | 4 | q35.2 | 190909127 | - | - | 0.64 | 0.2 | 1.29 | - | - | 13.15 | - | 5.66 | 7.31 | 2.17 | 1.22 | 30.42 |
|  | 175 | 4 | p11 | 49658600 | 1.11 | - | 4.31 | 28 | 2.58 | - | 3.22 | - | - | - | 2.43 | 4.89 | 3.73 | 46.54 |
|  | 176 | 5 | p15.33 | 717367 | 7.77 | 3.33 | 0.64 | 0.2 | 3.22 | - | - | 2.63 | - | - | - | 1.63 | 1.34 | 19.42 |
|  | 177 | 5 | q13.2 | 69233415 | - | - | 4.09 | 1.45 | 1.29 | - | 3.22 | 2.63 | - | - | 2.43 | 1.08 | 1.92 | 16.19 |
|  | 178 | 5 | q13.2 | 70254883 | 1.11 | - | 2.37 | 1.25 | - | - | 3.22 | - | 2.22 | 7.54 | - | 0.54 | 1.45 | 18.25 |
|  | 179 | 7 | q11.21 | 61728395 | 1.11 | - | 0.43 | 0.41 | 1.29 | - | - | 5.26 | - | - | 4.87 | 3.26 | 0.99 | 16.63 |
|  | 180 | 7 | q11.21 | 61987161 | 1.11 | - | 7.11 | 5.83 | 0.64 | - | 3.22 | - | - | 7.54 | - | 1.08 | 4.08 | 26.53 |
|  | 181 | 7 | q11.21 | 62156326 | - | - | 1.07 | 0.62 | - | - | 3.22 | 5.26 | - | 1.88 | 2.43 | 1.08 | 0.87 | 15.56 |
|  | 182 | 8 | p23.1 | 7029978 | 1.11 | 3.33 | 1.07 | 1.45 | - | - | 6.45 | 2.63 | - | - | - | 2.17 | 3.73 | 18.21 |
|  | 183 | 8 | p23.1 | 7297328 | 1.11 | - | 2.37 | 6.25 | 3.22 | 2.27 | - | - | - | - | 2.43 | 0.54 | 2.91 | 18.19 |
|  | 184 | 8 | p23.1 | 12232269 | 2.22 | 1.11 | 0.21 | 0.2 | 1.93 | - | - | 2.63 | 2.22 | - | - | - | 0.58 | 10.52 |
|  | 185 | 8 | q11.1 | 46847522 | - | - | 13.1 | 11.87 | 0.64 | - | 29.03 | - | - | 24.5 | 14.63 | 2.71 | 8.86 | 96.48 |
|  | 186 | 8 | q11.1 | 47012218 | - | - | 14.43 | 16.66 | 3.87 | - | 12.9 | - | - | 5.66 | 24.39 | 24.45 | 12.53 | 102.36 |
|  | 187 | 9 | p24.3 | 46587 | - | - | 1.29 | 9.58 | 1.93 | - | 3.22 | - | - | 3.77 | 4.87 | 2.17 | 3.73 | 26.83 |
|  | 188 | 9 | p12 | 41692305 | - | - | 1.5 | 2.7 | 1.29 | - | 12.9 | 5.26 | - | 1.88 | - | 1.08 | 1.8 | 26.61 |
|  | 189 | 9 | p11.2 | 43654127 | 2.22 | - | 0.86 | 1.25 | 3.87 | - | - | 2.63 | - | 1.88 | - | 1.63 | 3.73 | 14.34 |
|  | 190 | 9 | p11.2 | 44244868 | - | - | 1.5 | 1.66 | 0.64 | - | - | 2.63 | - | 3.77 | 4.87 | 0.54 | 1.28 | 15.61 |
|  | 191 | 9 | p11.2 | 44396687 | - | - | 1.72 | 1.66 | 0.64 | - | - | - | 2.22 | 1.88 | 2.43 | 1.08 | 1.28 | 11.63 |
|  | 192 | 9 | p11.2 | 44424439 | 1.11 | 1.11 | 3.23 | 4.79 | 3.87 | - | - | - | 2.22 | - | - | 5.97 | 3.38 | 22.3 |
|  | 193 | 9 | p11.2 | 44446124 | - | - | 2.58 | 2.7 | 2.58 | - | 6.45 | 10.52 | - | 3.77 | - | 2.71 | 2.44 | 31.31 |
|  | 194 | 9 | p11.2 | 45534834 | 1.11 | - | 1.93 | 1.66 | 1.29 | - | 3.22 | - | - | - | 4.87 | 0.54 | 1.39 | 14.62 |
|  | 195 | 9 | p11.2 | 45723022 | - | - | 2.8 | 3.75 | 0.64 | - | - | 2.63 | - | 5.66 | 2.43 | 2.17 | 2.39 | 20.08 |
|  | 196 | 9 | q12 | 65672595 | - | - | 0.64 | 0.2 | 0.64 | - | 6.45 | 2.63 | - | - | 2.43 | 4.34 | 0.99 | 17.33 |
|  | 197 | 9 | q21.11 | 69062418 | - | - | 0.43 | 1.25 | - | - | 3.22 | 2.63 | 2.22 | - | 7.31 | 10.86 | 1.98 | 27.92 |
|  | 198 | 9 | q13 | 69978010 | - | - | 0.21 | 0.41 | - | - | - | 5.26 | 2.22 | 1.88 | 4.87 | 0.54 | 0.58 | 15.39 |
|  | 199 | 9 | p12 | 43800174 | - | - | 0.86 | 0.41 | - | - | 3.22 | - | 2.22 | 3.77 | 2.43 | 2.17 | 0.87 | 15.08 |
|  | 200 | 9 | p13.1 | 41484890 | - | 1.11 | 1.72 | 2.29 | 1.93 | - | 3.22 | - | - | - | 2.43 | 1.63 | 1.63 | 14.33 |
|  | 201 | 10 | q11.21 | 42525112 | - | - | 1.5 | 4.37 | 0.64 | - | 9.67 | 2.63 | - | - | 2.43 | 5.43 | 2.56 | 26.67 |
|  | 202 | 10 | q11.21 | 42614424 | - | - | 0.43 | 1.25 | 3.22 | - | 3.22 | 5.26 | - | - | 26.82 | 12.5 | 2.91 | 52.7 |
|  | 203 | 10 | q11.22 | 47062299 | 2.22 | - | 0.64 | 0.62 | 0.64 | - | - | 5.26 | - | - | 4.87 | 7.6 | 1.57 | 21.85 |
|  | 204 | 13 | q11 | 19045720 | - | 2.22 | 1.72 | 5.83 | 1.29 | - | - | - | - | 1.88 | 9.75 | 3.8 | 3.03 | 26.49 |
|  | 205 | 14 | q11.2 | 19335739 | - | 1.11 | 3.44 | 2.08 | 1.93 | - | 3.22 | - | - | 13.2 | - | 0.54 | 2.27 | 25.52 |
|  | 206 | 14 | q11.2 | 19382197 | 3.33 | 2.22 | 1.5 | 2.5 | 2.58 | - | - | 2.63 | - | - | - | 3.8 | 2.09 | 18.56 |
|  | 207 | 14 | q11.2 | 19416179 | 1.11 | 1.11 | 0.43 | 0.83 | 0.64 | - | - | 2.63 | - | - | - | 1.08 | 0.69 | 7.83 |
|  | 208 | 14 | q11.2 | 19689305 | - | 1.11 | 0.21 | 0.2 | 0.64 | 2.27 | - | - | - | 3 | - | 1.08 | 0.58 | 8.51 |
|  | 209 | 14 | q32.33 | 106078690 | 1.11 | 2.22 | 0.64 | 0.41 | 10.96 | 2.27 | - | - | 4.44 | - | - | 1.63 | 1.8 | 23.68 |
|  | 210 | 14 | q32.33 | 106224342 | - | - | 9.05 | 8.12 | 1.93 | - | 3.22 | 7.89 | - | 3.77 | - | 2.17 | 5.48 | 36.15 |
|  | 211 | 14 | q32.33 | 106821724 | - | 1.11 | 1.07 | 1.45 | 0.64 | - | 3.22 | - | - | 5.66 | - | 0.54 | 1.1 | 13.69 |
|  | 212 | 14 | q32.33 | 106777331 | 3.33 | 1.11 | - | 0.62 | 0.64 | 4.54 | - | - | - | 1.88 | - | 1.08 | 0.75 | 13.2 |
|  | 213 | 14 | q32.33 | 106342305 | - | - | 21.76 | 32.08 | 14.83 | - | 12.9 | 7.89 | - | 54.71 | - | 5.43 | 18.89 | 149.6 |
|  | 214 | 15 | q11.1 | 20262224 | 3.33 | 7.77 | 1.93 | 2.5 | 3.87 | - | - | 7.89 | - | - | - | 5.43 | 2.91 | 32.72 |
|  | 215 | 15 | q11.1 | 20472839 | - | 1.11 | 0.21 | 0.62 | 1.93 | 2.27 | 3.22 | - | - | - | - | 0.54 | 0.64 | 9.9 |
|  | 216 | 15 | q11.1 | 20590015 | 1.11 | 2.22 | 1.72 | 2.91 | 4.51 | - | - | - | 2.22 | - | - | 3.8 | 2.33 | 18.49 |
|  | 217 | 15 | q11.2 | 22042506 | 1.11 | - | 0.64 | 1.25 | 0.64 | 2.27 | - | - | 6.66 | 1.88 | - | - | 0.93 | 14.45 |
|  | 218 | 15 | q13.3 | 32458661 | 2.22 | - | 1.72 | 0.2 | - | 2.27 | 3.22 | - | 2.22 | 1.88 | - | - | 0.87 | 13.73 |
|  | 219 | 15 | q11.1 | 20578409 | - | - | 6.25 | 3.95 | 0.64 | - | 9.67 | 10.52 | - | 9.43 | - | 14.13 | 5.07 | 54.59 |
|  | 220 | 15 | q11.1 | 22285757 | 1.11 | - | 0.21 | 0.62 | 0.64 | - | 9.67 | - | 2.22 | - | - | 1.08 | 0.69 | 15.55 |
|  | 221 | 15 | q11.1 | 22673387 | - | - | 3.01 | 2.5 | 1.29 | - | 9.67 | - | - | 1.88 | 2.43 | 0.54 | 1.98 | 21.32 |
|  | 222 | 15 | q13.2 | 30821746 | 3.33 | 2.22 | 2.37 | 0.2 | 1.93 | 2.27 | - | - | - | - | - | 2.17 | 1.45 | 14.49 |
|  | 223 | 16 | p11.2 | 32244461 | 2.22 | - | 0.21 | 0.62 | 1.29 | - | - | - | 2.22 | - | 2.43 | 1.08 | 0.69 | 10.07 |
|  | 224 | 16 | p11.2 | 32395607 | - | 4.44 | 1.07 | 1.04 | 0.64 | 2.27 | - | - | 2.22 | - | - | 0.54 | 1.04 | 12.22 |
|  | 225 | 16 | p11.2 | 33447256 | - | 1.11 | 1.93 | 1.66 | 1.93 | - | - | - | - | 1.88 | 14.63 | 3.26 | 1.98 | 26.4 |
|  | 226 | 16 | p11.2 | 32180774 | - | - | 2.8 | 1.45 | 1.29 | 4.54 | - | - | - | 1.88 | 2.43 | 1.08 | 1.63 | 15.47 |
|  | 227 | 16 | p11.2 | 32743469 | - | - | 3.23 | 6.45 | 0.64 | - | 3.22 | - | - | 1.88 | 17.07 | 4.34 | 3.73 | 36.83 |
|  | 228 | 16 | p11.2 | 33097410 | 1.11 | - | - | 0.2 | 1.93 | 2.27 | - | - | 2.22 | - | 2.43 | 1.08 | 0.58 | 11.24 |
|  | 229 | 16 | p11.2 | 33631818 | 1.11 | 2.22 | - | - | 1.29 | - | - | - | 2.22 | 5.66 | 2.43 | 1.63 | 0.75 | 16.56 |
|  | 230 | 17 | q12 | 34437116 | 7.77 | - | - | - | 1.29 | 2.27 | 3.22 | 2.63 | 2 | - | 2.43 | - | 0.87 | 21.61 |
|  | 231 | 21 | q11.2 | 14452850 | - | - | 0.21 | 0.41 | 1.93 | - | 3.22 | 2.63 | - | - | 4.87 | 5.97 | 1.22 | 19.24 |
|  | 232 | 22 | q11.1 | 16055207 | - | - | 0.21 | 1.04 | 0.64 | - | 3.22 | - | - | 1.88 | 9.75 | 3.26 | 1.1 | 20 |
|  | 233 | 22 | q11.21 | 21567219 | 1.11 | 2.22 | - | - | 0.64 | - | - | 2.63 | - | 1.88 | 9.75 | 0.54 | 0.64 | 18.77 |
|  | 234 | 22 | q11.22 | 22386434 | 1.11 | 2.22 | 0.64 | 0.41 | 1.93 | - | - | 2.63 | - | - | - | 0.54 | 0.75 | 9.48 |
|  | 235 | 22 | q11.22 | 22943462 | 1.11 | - | 4.52 | 5.6 | 1.29 | - | - | 2.63 | - | 9 | - | 1.08 | 3.67 | 25.23 |
|  | 236 | 22 | q11.22 | 22997799 | - | - | 10.12 | 10 | 4.51 | - | 9.67 | 2.63 | - | - | 26.82 | 21.19 | 9.07 | 84.94 |
|  | 237 | 22 | q11.22 | 23046123 | 1.11 | 1.11 | 3.44 | 7.29 | 4.51 | - | 3.22 | - | - | - | - | 1.63 | 3.73 | 22.31 |
|  | 238 | X | p22.33 | 169805 | - | - | 1.07 | 0.41 | 0.64 | 2.27 | 3.22 | - | - | 1.88 | - | 1.63 | 0.81 | 11.12 |
|  | 239 | X | q21.31 | 88467849 | - | 1.11 | 5.81 | 10 | 7.74 | - | - | - | 2.22 | - | 14.63 | 3.26 | 5.88 | 44.77 |
|  | 240 | X | q21.31 | 89001630 | - | - | 4.31 | 5 | 1.29 | - | 9.67 | - | - | 1.88 | 7.31 | 9.78 | 4.13 | 39.24 |
|  | 241 | X | q21.31 | 89623611 | 2.22 | - | 0.86 | 0.41 | 3.22 | - | 3.22 | - | - | 5.66 | 12.19 | - | 1.28 | 27.78 |
|  | 242 | X | q21.31 | 91175204 | - | - | 3.87 | 8.75 | 4.51 | - | 19.35 | - | - | 5.66 | 7.31 | 0.54 | 4.66 | 49.99 |
|  | 243 | X | q21.31 | 92056886 | - | - | 10.34 | 7.7 | 1.29 | - | 3.22 | - | - | 32.07 | 2.43 | 4.89 | 6.7 | 61.94 |
|  | 244 | Y | p11.2 | 4181582 | - | - | 0.21 | 0.62 | 1.93 | - | 3.22 | 5.26 | 2.22 | - | - | 0.54 | 0.69 | 14 |
|  | 245 | Y | p11.2 | 5785892 | - | - | 0.21 | 0.2 | 1.93 | 2.27 | 3.22 | - | 4.44 | - | - | 1.08 | 0.64 | 13.35 |
|  | 246 | Y | p11.2 | 5826911 | 1.11 | 6.66 | 3.87 | 3.54 | 9.03 | 9.09 | - | - | - | - | - | 1.63 | 3.67 | 34.93 |
|  | 247 | Y | p11.2 | 5580798 | 1.11 | 3.33 | 1.07 | 1.45 | 2.58 | - | - | - | 4.44 | - | - | 0.54 | 1.34 | 14.52 |
|  | 248 | 1 | p36.33 | 98590 | - | - | 1.93 | 0.62 | - | - | - | 2.63 | - | 1.88 | 12.19 | 8.15 | 1.98 | 27.4 |
|  | 249 | 1 | p36.21 | 12852748 | - | 1.11 | 0.21 | - | 1.93 | - | 3.22 | - | 4.44 | 1.88 | - | - | 0.49 | 12.79 |
|  | 250 | 1 | p36.13 | 16819621 | - | - | - | 0.2 | 0.64 | - | 3.22 | 2.63 | - | - | 4.87 | 2.71 | 0.64 | 14.27 |
|  | 251 | 1 | p36.13 | 16871266 | 3.33 | - | 0.21 | 0.41 | 0.64 | - | - | 2.63 | - | - | - | 1.08 | 0.58 | 8.3 |
|  | 252 | 1 | q21.1 | 144007037 | - | - | 0.86 | 1.87 | 0.64 | - | 3.22 | 13.15 | - | - | - | 0.54 | 1.22 | 20.28 |
|  | 253 | 1 | q21.2 | 148627557 | - | - | 1.72 | 2.08 | 2.58 | - | 6.45 | 2.63 | - | - | - | 0.54 | 1.51 | 16 |
|  | 254 | 1 | q21.1 | 144337917 | - | - | 1.93 | 1.45 | 1.29 | - | - | 2.63 | - | - | 2.43 | 1.63 | 1.34 | 11.36 |
|  | 255 | 1 | q21.1 | 144471989 | - | - | 1.29 | 2.29 | - | - | - | 2.63 | - | 3.77 | 4.87 | 0.54 | 1.34 | 15.39 |
|  | 256 | 1 | q43 | 243258051 | - | - | 0.43 | - | 3.22 | - | 3.22 | 2.63 | - | - | 4.87 | 9.23 | 1.63 | 23.6 |
|  | 257 | 1 | p36.33 | 98590 | - | - | 1.93 | 0.62 | - | - | - | 2.63 | - | 1.88 | 12.19 | 8.15 | 1.98 | 27.4 |
|  | 258 | 1 | p36.21 | 12852748 | - | 1.11 | 0.21 | - | 1.93 | - | 3.22 | - | 4.44 | 1.88 | - | - | 0.52 | 12.79 |
|  | 259 | 1 | p36.13 | 16819621 | - | - | - | 0.2 | 0.64 | - | 3.22 | 2.63 | - | - | 4.87 | 2.71 | 0.64 | 14.27 |
| **6 Populations** | 260 | 1 | p36.13 | 16871266 | 3.33 | - | 0.21 | 0.41 | 0.64 | - | - | 2.63 | - | - | - | 1.08 | 0.58 | 8.3 |
|  | 261 | 2 | p11.2 | 89126133 | - | - | 16.37 | 8.33 | 1.29 | - | 22.58 | - | - | 3.77 | - | 1.63 | 7.58 | 53.97 |
|  | 262 | 2 | p11.2 | 89133619 | - | - | 0.43 | 1.45 | 7.74 | - | - | - | - | 3.77 | 9.75 | 13.04 | 2.97 | 36.18 |
|  | 263 | 2 | p11.2 | 89972669 | 1.11 | - | 0.21 | 0.2 | 0.64 | - | - | - | - | 1.88 | - | 0.54 | 0.4 | 4.58 |
|  | 264 | 2 | p11.2 | 91645185 | - | - | 0.64 | 0.41 | 0.64 | - | 3.22 | 10.52 | - | - | - | 2.71 | 0.93 | 18.14 |
|  | 265 | 2 | p11.1 | 91921793 | - | - | 0.43 | 0.41 | 0.64 | - | 6.45 | 10.52 | - | - | - | 4.89 | 1.16 | 23.34 |
|  | 266 | 2 | q13 | 111084885 | - | - | 0.86 | 1.25 | 1.29 | 2.27 | - | - | - | - | 2.43 | 2.17 | 1.04 | 10.27 |
|  | 267 | 2 | p11.2 | 90195665 | - | - | 1.29 | 2.29 | 3.87 | - | - | 2.63 | - | - | 29.26 | 3.8 | 2.5 | 43.14 |
|  | 268 | 2 | p11.1 | 91653350 | - | - | 0.43 | - | 3.22 | - | 3.22 | 7.89 | - | - | 31.7 | 9.23 | 2.39 | 55.69 |
|  | 269 | 2 | p11.2 | 89593743 | - | - | 1.72 | 0.2 | 1.93 | - | 3.22 | - | - | - | 2.43 | 0.54 | 0.87 | 10.04 |
|  | 270 | 2 | p11.2 | 90211593 | - | - | 1.72 | 0.62 | 0.64 | - | - | 2.63 | - | 1.88 | - | 2.71 | 1.1 | 10.2 |
|  | 271 | 2 | q37.3 | 242915454 | - | 3.33 | 10.34 | 10 | - | - | - | 7.89 | - | 5.66 | - | 5.97 | 6.76 | 43.19 |
|  | 272 | 3 | p12.3 | 75427095 | - | 6 | 1.07 | 1.45 | - | 2.27 | - | - | - | 7.54 | - | 4.34 | 1.8 | 22.67 |
|  | 273 | 3 | p12.3 | 75500752 | - | - | 0.86 | 0.41 | 0.64 | - | 6.45 | - | - | - | 6 | 8.69 | 1.8 | 23.05 |
|  | 274 | 4 | p11 | 49590590 | - | - | 0.43 | 1.87 | 0.64 | - | 3.22 | 5.26 | - | - | - | 4.34 | 1.34 | 15.76 |
|  | 275 | 4 | p16.1 | 9370690 | 8.88 | - | - | 0.2 | 0.64 | - | - | - | 2.22 | - | 2.43 | 2.71 | 0.99 | 17.08 |
|  | 276 | 4 | q13.2 | 70123498 | - | - | 3.23 | 3.75 | 2.58 | - | - | 2.63 | - | - | 2.43 | 7.06 | 3.03 | 21.68 |
|  | 277 | 4 | q35.2 | 190822375 | - | - | 0.86 | 0.41 | 1.29 | - | 6.45 | - | - | 1.88 | - | 2.17 | 0.87 | 13.06 |
|  | 278 | 4 | q35.2 | 190899664 | - | - | 0.64 | 0.41 | 1.29 | - | - | - | - | 1.88 | 4.87 | 4.89 | 1.1 | 13.98 |
|  | 279 | 4 | q35.2 | 190911343 | - | - | 0.43 | 0.83 | 1.29 | - | - | 5.26 | - | 1.88 | - | 0.54 | 0.69 | 10.23 |
|  | 280 | 4 | q13.2 | 69485967 | - | - | 1.29 | 1.25 | - | - | 3.22 | - | - | 7.54 | 2.43 | 2.17 | 1.28 | 17.9 |
|  | 281 | 4 | q13.2 | 69505103 | - | - | 7.97 | 61.25 | - | - | 16.12 | 2.63 | - | - | 4.87 | 1.08 | 4.48 | 93.92 |
|  | 282 | 7 | q35 | 143911612 | 3.33 | 1.11 | 1.72 | 2.7 | - | - | - | 5.26 | - | - | - | 2.71 | 1.86 | 16.83 |
|  | 283 | 7 | q35 | 143927795 | 3.33 | 1.11 | - | 0.2 | 0.64 | - | - | - | - | 1.88 | - | 0.54 | 0.46 | 7.7 |
|  | 284 | 8 | p23.1 | 6999220 | - | 1.11 | - | 0.2 | - | - | 3.22 | 2.63 | - | - | 2.43 | 0.54 | 0.34 | 10.13 |
|  | 285 | 8 | p23.1 | 7267353 | - | 1.11 | 1.72 | 0.41 | 1.29 | 9.09 | - | - | - | - | - | 1.08 | 1.1 | 14.7 |
|  | 286 | 8 | p11.22 | 39230313 | - | - | 0.86 | 1.45 | 1.29 | - | 6.45 | - | - | - | 9.75 | 5.43 | 1.69 | 25.23 |
|  | 287 | 8 | p11.22 | 39248730 | - | 1.11 | 0.21 | 0.2 | - | 4.54 | - | 5.26 | - | - | - | 1.08 | 0.52 | 12.4 |
|  | 288 | 8 | q11.1 | 47000863 | - | - | 9.26 | 9.5 | 0.64 | - | 6.45 | - | - | 9.43 | - | 7.06 | 6.41 | 42.34 |
|  | 289 | 8 | q11.1 | 47009583 | - | - | 2.37 | 0.41 | 2.58 | - | 3.22 | - | - | 1.88 | - | 3.08 | 1.51 | 13.54 |
|  | 290 | 8 | q24.23 | 137681619 | - | 6 | 3.01 | 3.75 | - | - | - | 3 | - | 1.88 | - | 4.89 | 2.97 | 22.53 |
|  | 291 | 8 | p23.1 | 7783150 | - | - | 0.64 | 1.66 | - | - | 3.22 | - | - | 1.88 | 4.87 | 1.08 | 0.99 | 13.35 |
|  | 292 | 8 | q11.1 | 46912015 | - | - | 7.54 | 12.29 | 1.29 | - | - | - | - | 11.32 | 7.31 | 0.54 | 6.12 | 40.29 |
|  | 293 | 9 | p13.1 | 40911212 | - | - | 1.07 | 0.83 | 1.29 | - | - | 2.63 | - | - | 2.43 | 2.71 | 1.04 | 10.96 |
|  | 294 | 9 | p12 | 41475094 | 3.33 | - | 0.43 | 0.41 | - | - | 3.22 | - | 2.22 | - | 2.43 | - | 0.58 | 12.04 |
|  | 295 | 9 | q13 | 68683835 | - | - | 2.15 | 4.58 | 5.16 | - | - | - | 2.22 | - | 2.43 | 2.17 | 2.68 | 18.71 |
|  | 296 | 9 | p13.1 | 44469316 | - | 1.11 | 1.72 | 0.83 | 0.64 | - | - | 5.26 | - | - | - | 1.63 | - | 11.19 |
|  | 297 | 9 | p11.2 | 44855725 | - | 1.11 | 0.64 | 1.04 | 1.29 | - | - | - | - | - | 2.43 | 0.54 | 0.75 | 7.05 |
|  | 298 | 9 | p11.2 | 45448932 | - | - | 0.86 | 0.83 | - | - | - | 2.63 | - | 5.66 | 2.43 | 2.17 | 0.99 | 14.58 |
|  | 299 | 9 | q13 | 67360675 | - | - | 2.37 | 0.83 | 1.93 | - | - | 5.26 | - | 3.77 | - | 0.54 | 1.34 | 14.7 |
|  | 300 | 9 | p13.1 | 42136014 | - | - | 0.21 | 0.62 | 0.64 | - | 3.22 | - | 2.22 | - | - | 0.54 | 0.46 | 7.45 |
|  | 301 | 10 | q11.22 | 46691159 | - | 1.11 | 1.29 | 0.62 | - | - | - | 2.63 | 2.22 | - | - | 3.8 | 1.1 | 11.67 |
|  | 302 | 10 | q11.22 | 46918172 | - | 5 | 12.71 | 13.5 | - | - | - | - | - | 1.88 | 2.43 | 5.97 | 8.27 | 41.49 |
|  | 303 | 10 | q11.22 | 48714571 | - | - | 3.87 | 1.45 | - | - | - | 2.63 | - | 5.66 | 34.39 | 6.52 | 2.97 | 54.52 |
|  | 304 | 10 | q11.22 | 48883390 | 1.11 | 2 | 0.86 | 2.5 | - | - | 3.22 | - | - | - | - | 0.54 | 1.22 | 10.23 |
|  | 305 | 10 | q11.23 | 51197126 | - | - | 2.58 | 3.54 | 1.29 | - | - | 2.63 | - | 1.88 | - | 2.17 | 2.15 | 14.09 |
|  | 306 | 10 | q26.3 | 135242873 | 7.77 | 2.22 | 2.37 | 1.66 | 1.29 | - | - | - | 2.22 | 1.88 | - | - | - | 19.41 |
|  | 307 | 11 | p15.4 | 4256117 | 3.33 | - | 0.64 | 1.45 | 1.29 | - | 3.22 | - | - | - | - | 0.54 | 0.99 | 10.47 |
|  | 308 | 12 | p13.31 | 8343961 | - | - | 1.5 | 1.45 | 0.64 | - | 3.22 | - | - | - | 12.19 | 5.43 | 1.8 | 24.43 |
|  | 309 | 14 | q11.2 | 19802529 | - | 2 | 3.44 | 1.25 | 0.64 | 2.27 | - | - | - | - | - | 1.63 | 1.69 | 11.23 |
|  | 310 | 14 | q11.2 | 20010901 | 1.11 | - | 0.21 | 1.25 | 1.93 | - | - | 2.63 | - | - | - | 2.71 | 0.99 | 9.84 |
|  | 311 | 14 | q11.2 | 20315192 | - | - | 0.43 | 0.41 | 0.64 | - | - | 2.63 | - | - | 2.43 | 0.54 | 0.46 | 7.08 |
|  | 312 | 14 | q32.33 | 106054885 | 1.11 | - | 0.43 | - | 2.58 | - | 6.45 | - | 2.22 | 1.88 | - | - | 0.64 | 14.67 |
|  | 313 | 14 | q32.33 | 106295666 | - | - | 3.87 | 6.04 | 1.93 | - | 12.9 | - | - | - | 26.82 | 3.26 | 4.13 | 54.82 |
|  | 314 | 14 | q32.33 | 106547495 | 1.11 | 1.11 | 1.07 | 0.62 | - | 2.27 | - | - | - | - | - | 1.63 | 0.81 | 7.81 |
|  | 315 | 14 | q32.33 | 106820732 | 3.33 | 1.11 | 1.29 | 0.83 | - | - | 3.22 | - | - | - | - | 1.08 | 0.99 | 10.86 |
|  | 316 | 14 | q32.33 | 106825904 | - | - | 0.86 | 0.2 | 0.64 | - | 3.22 | - | - | 5.66 | - | 1.63 | 0.75 | 12.21 |
|  | 317 | 14 | q11.2 | 22880443 | - | - | 1.5 | 1.04 | 1.29 | - | - | 5.26 | - | 1.88 | - | 0.54 | 1.04 | 11.51 |
|  | 318 | 14 | q11.2 | 22960820 | - | - | 0.64 | 0.2 | 4.51 | - | 3.22 | 5.26 | - | - | - | 1.08 | 0.93 | 14.91 |
|  | 319 | 14 | q32.33 | 106448068 | - | - | 0.21 | 0.83 | 3.22 | - | - | 5.26 | - | - | 2.43 | 2.17 | 0.99 | 14.12 |
|  | 320 | 14 | q32.33 | 106526954 | - | - | 3.23 | 3.54 | 0.64 | - | - | 10.52 | - | - | 4.87 | 8.15 | 3.14 | 30.95 |
|  | 321 | 14 | q32.33 | 106872050 | 1.11 | 2.22 | 0.43 | 0.62 | - | - | - | 2.63 | - | 1.88 | - | - | 0.58 | 8.89 |
|  | 322 | 14 | q32.33 | 106934536 | - | 2.22 | 3.87 | 3.75 | - | - | - | - | - | 1.88 | 2.43 | 4.34 | 2.79 | 18.49 |
|  | 323 | 15 | q11.1 | 20440526 | - | - | 4.52 | 6.87 | 1.29 | - | - | - | 6.66 | 3.77 | - | 1.08 | 3.67 | 24.19 |
|  | 324 | 15 | q11.1 | 20446126 | 4.44 | 1.11 | 0.43 | 1.04 | 0.64 | - | - | - | - | - | - | 0.54 | 0.81 | 8.2 |
|  | 325 | 15 | q11.2 | 21193866 | - | - | 1.72 | 2.08 | - | 2.27 | 3.22 | - | - | 1.88 | - | 1.63 | 1.39 | 12.8 |
|  | 326 | 15 | q11.2 | 21271038 | 2.22 | 1.11 | - | 0.62 | - | 4.54 | - | - | 6.66 | - | - | 1.08 | 0.75 | 16.23 |
|  | 327 | 15 | q11.2 | 21905745 | - | 2.22 | - | 0.41 | 1.29 | - | - | - | 2.22 | 1.88 | - | 2.71 | 0.75 | 10.73 |
|  | 328 | 15 | q11.2 | 22350985 | - | - | 5.81 | 7.5 | 0.64 | - | - | 2.63 | - | 3.77 | - | 1.08 | 4.02 | 21.43 |
|  | 329 | 15 | q11.2 | 22384989 | - | 2.22 | 2.37 | 1.66 | - | - | - | 2.63 | - | 3.77 | - | 2.17 | 1.63 | 14.82 |
|  | 330 | 15 | q11.2 | 24512023 | 1.11 | - | 1.5 | 2.29 | - | - | - | - | 2.22 | - | 2.43 | 1.63 | 1.39 | 11.18 |
|  | 331 | 15 | q25.2 | 84825273 | - | - | 3.01 | 8.75 | - | - | 9.67 | - | - | 1.88 | 2.43 | 3.8 | 3.96 | 29.54 |
|  | 332 | 15 | q11.1 | 22673387 | - | - | 3.01 | 2.5 | 1.29 | - | 9.67 | - | - | - | 2.43 | 0.54 |  | 19.44 |
|  | 333 | 16 | p13.11 | 16628050 | - | - | 0.21 | 0.41 | 3.22 | 2.27 | - | - | 2.22 | - | - | 0.54 | 0.64 | 8.87 |
|  | 334 | 16 | p12.3 | 18556708 | - | - | 1.07 | 0.62 | 1.29 | - | 3.22 | - | 4.44 | - | - | 1.63 | 0.93 | 12.27 |
|  | 335 | 16 | p12.3 | 18594388 | - | 1.11 | 0.43 | - | 0.64 | 4.54 | - | - | 2.22 | - | - | 1.08 | 0.52 | 10.02 |
|  | 336 | 16 | p12.2 | 21412391 | 1.11 | - | - | 0.2 | 1.29 | 2.27 | - | - | 6.66 | - | - | 0.54 | 0.52 | 12.07 |
|  | 337 | 16 | p11.2 | 32743469 | 1.11 | - | 1.07 | 0.2 | 1.29 | - | 6.45 | - | - | - | - | 2.17 | 0.87 | 12.29 |
|  | 338 | 16 | p11.2 | 33452725 | - | - | 0.86 | 1.87 | - | - | 3.22 | - | - | 5.66 | 2.43 | 1.08 | 1.16 | 15.12 |
|  | 339 | 16 | p11.2 | 34466559 | - | 16.66 | 4.09 | 6.25 | - | - | - | - | - | 18.86 | 2.43 | 10.32 | 5.48 | 58.61 |
|  | 340 | 16 | p13.11 | 16798651 | - | - | 0.21 | - | 1.93 | - | 3.22 | - | 2.22 | - | 2.43 | 2.17 | 0.64 | 12.18 |
|  | 341 | 16 | p11.2 | 33811489 | - | - | 1.5 | 1.04 | - | - | 3.22 | 7.89 | 2.22 | - | - | 1.08 | 1.1 | 16.95 |
|  | 342 | 17 | q21.31 | 44214888 | - | 10 | 11.63 | 13.12 | 0.64 | - | - | - | - | 5.66 | - | 3.8 | 7.98 | 44.85 |
|  | 343 | 17 | q21.31 | 44344847 | - | - | 1.29 | 1.66 | - | - | 3.22 | - | - | 1.88 | 2.43 | 3.8 | 1.39 | 14.28 |
|  | 344 | 17 | p11.2 | 18283285 | 1.11 | 4.44 | 5.17 | 5.41 | 2.58 | - | - | - | - | - | - | 5.97 | 4.08 | 24.68 |
|  | 345 | 17 | q21.31 | 44156069 | - | 13.33 | 10.77 | 12.08 | 0.64 | - | - | 13.15 | - | - | - | 3.26 | 7.69 | 53.23 |
|  | 346 | 18 | p11.21 | 15401751 | - | - | 1.07 | 1.87 | 0.64 | - | - | 2.63 | - | - | 24.39 | 7.06 | 2.27 | 37.66 |
|  | 347 | 19 | p12 | 20598431 | - | - | 0.21 | 0.2 | 1.29 | 6.81 | - | - | 2.22 | - | - | 0.54 | 0.52 | 11.27 |
|  | 348 | 19 | p12 | 20603318 | - | 1.11 | 0.43 | 0.2 | 1.93 | 2.27 | 9.67 | - | - | - | - | - | 0.64 | 15.61 |
|  | 349 | 19 | q13.2 | 43321125 | - | - | 5.6 | 5.2 | - | 2.27 | 3.22 | 2.63 | - | - | - | 0.54 | 3.2 | 19.46 |
|  | 350 | 19 | q13.2 | 43542819 | - | - | 1.07 | 0.2 | 0.64 | - | 3.22 | 2.63 | - | - | - | 2.17 | 0.75 | 9.93 |
|  | 351 | 21 | p11.1 | 11173781 | - | 1.11 | 4.95 | 2.08 | 2.58 | - | - | 15.78 | - | - | - | 4.89 | 3.09 | 31.39 |
|  | 352 | 22 | q11.21 | 21845282 | 1.11 | 1.11 | 0.64 | - | 1.29 | - | - | - | - | - | 2.43 | 1.08 | 0.58 | 7.66 |
|  | 353 | 22 | q11.22 | 23258994 | - | - | 1.07 | 2.11 | - | 2.27 | - | 7.89 | - | - | 2.43 | 2.17 | 1.69 | 17.94 |
|  | 354 | 22 | q13.32 | 49045400 | - | - | 0.86 | 3.54 | - | - | 3.22 | - | - | 1.88 | 2.43 | 1.63 | 1.57 | 13.56 |
|  | 355 | 22 | q11.21 | 21607072 | 1.11 | - | 0.21 | 0.41 | 0.64 | - | - | 5.26 | - | 1.88 | - | - | 0.46 | 9.51 |
|  | 356 | 22 | q11.22 | 22871748 | - | - | 1.07 | 0.83 | 0.64 | - | 3.22 | - | - | 3.77 | - | 0.54 | 0.81 | 10.07 |
|  | 357 | 22 | q11.22 | 22962462 | - | - | 3.66 | 2.7 | 1.29 | - | 19.35 | - | - | 7.54 | - | 8.15 | 3.32 | 42.69 |
|  | 358 | 22 | q11.22 | 23051908 | - | - | 2.58 | 5.83 | 5.16 | - | - | - | - | 15.09 | 2.43 | 1.08 | 3.44 | 32.17 |
|  | 359 | 22 | q11.22 | 23078678 | - | - | 0.43 | 4.37 | 3.87 | - | 3.22 | 7.89 | - | - | - | 3.8 | 2.33 | 23.58 |
|  | 360 | 22 | q11.22 | 23112739 | - | - | 4.31 | 0.83 | 0.64 | - | 6.45 | - | - | 11.32 | - | 0.54 | 1.98 | 24.09 |
|  | 361 | 22 | q11.22 | 23259859 | - | - | 1.07 | 6.87 | 3.87 | - | - | - | - | 1.88 | 2.43 | 2.71 | 2.97 | 18.83 |
|  | 362 | 22 | q11.23 | 24283004 | 1.11 | - | 0.43 | 0.2 | 3.22 | - | - | - | 2.22 | - | - | 1.63 | 0.75 | 8.81 |
|  | 363 | X | p11.23 | 47880145 | 2.22 | 1.11 | 1.07 | 0.2 | 1.93 | - | - | - | - | 1.88 | - | - | 0.75 | 8.41 |
|  | 364 | X | q21.1 | 76362280 | 3.33 | - | 1.29 | 0.83 | 0.64 | 2.27 | - | - | - | 1.88 | - | - | 0.93 | 10.24 |
|  | 365 | X | q21.31 | 88466435 | - | - | 2.15 | 4.79 | 4.51 | - | - | 5.26 | - | - | 34.14 | 3.26 | 3.61 | 54.11 |
|  | 366 | X | q21.31 | 88499792 | - | - | 1.29 | 0.41 | 1.29 | 2.27 | - | - | 2.22 | 3.77 | - | - | 0.81 | 11.25 |
|  | 367 | X | q21.31 | 88970028 | - | - | 1.5 | 1.66 | 3.22 | - | 6.45 | - | - | - | 34.14 | 8.15 | 2.97 | 55.12 |
|  | 368 | X | q21.31 | 89011979 | - | - | 1.29 | 5.2 | - | - | - | - | 2.22 | 5.66 | 9.75 | 5.97 | 2.91 | 30.09 |
|  | 369 | X | q21.31 | 89355671 | - | - | 19 | 4.58 | 0.64 | - | - | - | - | 7.54 | 2.43 | 0.54 | 2.79 | 34.73 |
|  | 370 | X | q21.31 | 89849761 | - | - | 1.29 | 4.16 | 1.29 | - | 6.45 | - | - | - | 2.43 | 1.08 | 1.92 | 16.7 |
|  | 371 | X | q21.31 | 91162118 | - | - | 1.5 | 4.58 | 1.93 | - | - | 2.63 | - | 3.77 | - | 1.63 | 2.21 | 16.04 |
|  | 372 | X | q21.31 | 91292916 | - | - | 1.93 | 2.08 | 1.93 | - | - | - | - | 1.88 | 2.43 | 1.08 | 1.51 | 11.33 |
|  | 373 | X | q21.31 | 91625154 | - | - | 1.72 | 1.25 | - | - | 3.22 | - | - | 1.88 | 2.43 | 1.08 | 1.1 | 11.58 |
|  | 374 | X | q21.32 | 91891109 | - | - | 0.43 | 0.41 | 0.64 | - | 3.22 | 2.63 | - | - | - | 1.08 | 0.52 | 8.41 |
|  | 375 | X | q21.32 | 91916478 | - | - | 2.15 | 1.45 | 1.93 | - | 3.22 | - | - | - | 2.43 | 1.63 | 1.45 | 12.81 |
|  | 376 | X | q21.32 | 91947527 | - | - | 0.64 | 2.5 | 3.87 | - | - | - | - | 1.88 | 16 | 7.6 | 3.03 | 32.49 |
|  | 377 | X | q24 | 119994797 | - | 1.11 | 1.29 | 0.62 | 3.87 | - | 6.45 | - | - | - | - | 1.08 | 1.16 | 14.42 |
|  | 378 | X | q21.31 | 90972200 | - | - | 2.8 | 2.08 | 4.51 | - | - | 2.63 | - | - | 4.87 | 0.54 | 1.98 | 17.43 |
|  | 379 | Y | p11.2 | 3352707 | - | 3.33 | 5.17 | 6.25 | 7.74 | - | 3.22 | - | - | - | - | 1.63 | 4.25 | 27.34 |
|  | 380 |  |  |  |  |  |  |  |  |  |  |  |  |  |  |  |  |  |
|  | 381 |  |  |  |  |  |  |  |  |  |  |  |  |  |  |  |  |  |
|  | 382 |  |  |  |  |  |  |  |  |  |  |  |  |  |  |  |  |  |
|  | 383 |  |  |  |  |  |  |  |  |  |  |  |  |  |  |  |  |  |
|  | 384 |  |  |  |  |  |  |  |  |  |  |  |  |  |  |  |  |  |
|  | 385 |  |  |  |  |  |  |  |  |  |  |  |  |  |  |  |  |  |
|  |  |  |  |  |  |  |  |  |  |  |  |  |  |  |  |  |  |  |
|  |  |  |  |  |  |  |  |  |  |  |  |  |  |  |  |  |  |  |
|  |  |  |  |  |  |  |  |  |  |  |  |  |  |  |  |  |  |  |
|  |  |  |  |  |  |  |  |  |  |  |  |  |  |  |  |  |  |  |
|  |  |  |  |  |  |  |  |  |  |  |  |  |  |  |  |  |  |  |
|  |  |  |  |  |  |  |  |  |  |  |  |  |  |  |  |  |  |  |
|  |  |  |  |  |  |  |  |  |  |  |  |  |  |  |  |  |  |  |
|  |  |  |  |  |  |  |  |  |  |  |  |  |  |  |  |  |  |  |
|  |  |  |  |  |  |  |  |  |  |  |  |  |  |  |  |  |  |  |
|  |  |  |  |  |  |  |  |  |  |  |  |  |  |  |  |  |  |  |
|  |  |  |  |  |  |  |  |  |  |  |  |  |  |  |  |  |  |  |
|  |  |  |  |  |  |  |  |  |  |  |  |  |  |  |  |  |  |  |
